# Integrated AUF-UPLC-MS and fraction-activity correlation to identify COX-2-targeted constituents of Shaoyao Gancao decoction

**DOI:** 10.3389/fchem.2026.1912431

**Published:** 2026-09-21

**Authors:** Liping Li, Jun Chi, Hejia Wan, Yadi Hou, Bingjie Ren, Yixiao Li, Liping Dai

**Affiliations:** 1 Henan University of Chinese Medicine, Henan Collaborative Innovation Center for Research and Development on the Whole Industry Chain of Yu-Yao, Zhengzhou, China; 2 Engineering Technology Research Center for Comprehensive Development and Utilization of Authentic Medicinal Materials in Henan Province, Henan University of Chinese Medicine, Zhengzhou, China

**Keywords:** affinity ultrafiltration, bioactive fractions, fraction-activity correlation, *in vitro* COX-2 inhibition, molecular dynamics simulation, potential COX-2 binding constituents, Shaoyao Gancao decoction, UPLC-MS

## Abstract

Shaoyao Gancao Decoction (SGD), a classical multi-ingredient herbal formula, exerts potent anti-inflammatory and anti-rheumatoid arthritis effects against rheumatoid arthritis (RA). Existing cellular experiments have proven that SGD can downregulate Cyclooxygenase-2 (COX-2) expression in inflammatory macrophages, implying a potential link between its anti-arthritic efficacy and COX-2 modulation. Nevertheless, its constituents with potential COX-2 binding capacity and corresponding anti-RA material basis are unclear, hindering its pharmaceutical exploitation. In this study, an integrated strategy combining affinity ultrafiltration-UPLC-MS screening, fraction-activity correlation modeling, molecular simulation and *in vitro* enzyme assays was constructed to characterize COX-2-interacting components from SGD. Nineteen COX-2-binding constituents were captured and characterized, mainly paeoniflorin, glycyrrhizic acid and licorice glycoside B. Derived from Bai Shao (BS) and Zhi Gancao (ZGC), these compounds cover monoterpenoid glycosides, triterpenoid saponins, flavonoids and fatty acids, embodying SGD’s multi-component feature. Molecular docking results predicted favorable binding interactions, and molecular dynamics simulations further demonstrated that core monomers could form stable COX-2 binding complexes. These core components stably bound COX-2 via persistent hydrogen bonds. *In vitro* enzyme tests confirmed paeoniflorin, albiflorin, glycyrrhizic acid and glycyrrhetinic acid strongly suppressed COX-2 with IC_50_ of 23.8, 53.2, 54.1 and 58.9 μM, respectively. Among serial gradient eluates (10%–70% gradient) of BS, the 30% ethanol fraction exhibited the most potent COX-2 inhibitory activity (IC_50_ = 27.63 μg/mL). Further enrichment via 40% ethanol macroporous separation yielded a refined BS fraction with superior activity (IC_50_ = 23.8 μg/mL). Cell assays on LPS-induced RAW264.7 macrophages indicated the reconstituted active fraction of Shaoyao Gancao Decoction (SGF) inhibited NO release more effectively than crude SGD extract. This study established a robust workflow coupling target screening, chemical identification, computational validation and active fraction enrichment for complex herbal extracts. This work screened potential COX-2-binding constituents from SGD using analytical chemistry workflows and verified the COX-2-inhibitory potency of four monomers via *in-vitro* enzyme assays. Prior cellular evidence supports that SGD suppresses COX-2 in LPS-stimulated macrophages, suggesting a link between its anti-inflammatory action and COX-2 modulation. However, systematic *in vivo* RA animal studies are needed to validate the COX-2-dependent anti-RA mechanism of SGD, which lies outside the analytical scope of the present investigation.

## 1 Introduction

Shaoyao Gancao Decoction (SGD), a canonical herbal formula originating from Treatise on Typhoid and Miscellaneous Diseases, has long been prescribed for pain and spasm disorders driven by yin-blood insufficiency (Qu et al., 2020). The formula was officially cataloged into China’s First Catalogue of Ancient Classical Formulas in 2018 (National Administration of Traditional Chinese Medicine, 2018), consisting of equal-mass Bai Shao (BS) and Zhi Gancao (ZGC). Clinically, SGD has been widely used to manage neck, shoulder, lumbar, and leg pain, gastrointestinal disorders, neurological diseases, and skin conditions (Gao et al., 2025). Multiple pharmacological investigations have validated the anti-rheumatoid arthritis (anti-RA) capacity of SGD, and computational predictions suggest its components may exert anti-inflammatory effects by interacting with multiple inflammation-related proteins (Chu, 2012; Ma et al., 2026). From a phytochemical perspective, SGD is abundant in monoterpene glycosides, flavonoids, triterpenoid saponins and phenolic acids, among which paeoniflorin, glycyrrhizic acid and liquiritin serve as signature phytochemical markers (Li et al., 2015). Paeoniflorin-enriched total glucosides of paeony have been clinically authorized for RA intervention owing to mild systemic toxicity and liver-protective effects (Yuan et al., 2016), while glycyrrhetinic acid also manifests potent anti-inflammatory and anti-allergic potency (Hu et al., 2020). Nevertheless, the exact COX-2-binding phytochemicals and bioactive fractions contributing to the analgesic and anti-inflammatory performance of SGD remain insufficiently characterized via targeted screening approaches.

Cyclooxygenase-2 (COX-2) acts as an inflammation-triggered rate-limiting enzyme that catalyzes arachidonic acid conversion into prostaglandin E_2_ (PGE_2_). Excessive COX-2 expression in RA lesions accumulates abundant PGE_2_, ultimately triggering inflammatory vasodilation, hyperalgesia and irreversible articular damage (Jiang et al., 2007; Xie et al., 2020). Conventional synthetic compounds with COX-2 binding capacity mitigate gastrointestinal toxicity relative to non-selective NSAIDs, yet their latent cardiovascular adverse effects restrict long-duration clinical administration (Shuai et al., 2025). There is therefore an urgent demand to exploit natural COX-2 antagonists from herbal matrices with superior biosafety profiles for inflammatory arthropathy therapy.

Herein, we constructed an integrated affinity ultrafiltration (AUF)-UPLC-MS platform for targeted mining and structural identification of COX-2 ligands from complex SGD extracts. partial-least-squares (PLS) regression analysis was adopted to construct fraction-activity correlation and pinpoint core bioactive constituents (Chen et al., 2024). The binding potentials of candidate compounds were predicted via molecular docking and molecular dynamics simulations, and their actual COX-2-suppressive activity was verified by subsequent *in vitro* enzyme inhibition assays. Furthermore, macroporous resin chromatography was implemented to fractionate crude SGD extracts, and the optimally mixed bioactive fraction (SGF) was assessed for anti-inflammatory efficacy using RAW 264.7 macrophage cell models. This work establishes a streamlined affinity-ultrafiltration-based screening strategy to discover potential bioactive molecules from complex herbal extracts and provides preliminary analytical evidence for further exploration of SGD-derived anti-inflammatory candidates.

## 2 Materials and methods

### 2.1 Materials and reagents

Vanquish™ UPLC ultra performance liquid chromatography (UPLC) system, coupled with an Orbitrap Exploris 120 high-resolution mass spectrometer (HRMS) (Thermo Scientific, United States of America); a Milli-Q water purification system (Milipore, United States of America); SpectraMax iD5 microplate reader (Shanghai Molecular Devices Instruments Co., Ltd., China); vortex mixer (Haimen Kylin-Bell Lab Instruments Co., Ltd., China); thermostat (metal bath) (Hangzhou Ruicheng Instrument Co., Ltd., China); and high-speed centrifuge (Thermo Scientific, United States of America).

The medicinal slices of BS (batch No. 20072013) and ZGC (batch No. 21042416) were purchased from Henan Zhangzhongjing Pharmacy Co., Ltd. The two herbs were authenticated by Professor Liping Dai from Henan University of Chinese Medicine: BS corresponds to the dried root of Paeonia lactiflora Pall. (Ranunculaceae), and ZGC corresponds to the dried root and rhizome of Glycyrrhiza uralensis Fisch. (Fabaceae). Voucher specimens of BS and ZGC were deposited in the Herbarium of Henan University of Chinese Medicine, and their quality complies with the standards recorded in the 2020 edition of Chinese Pharmacopoeia. Reference standards, including paeoniflorin (batch No. RDD-S01002304028) and albiflorin (batch No. RDD-S01002307004), were purchased from Chengdu Ruifensidan Biotechnology Co., Ltd. liquiritin (No. 220415) and glycyrrhizic acid (No. 220102) were purchased from Chengdu Herb Substance Biotechnology co., Ltd., all with purities ≥98%. The chromatographic grade formic acid, methanol and acetonitrile were purchased from Thermo Scientific, United States of America; DMSO was obtained from Tianjin Hengxing Chemical Reagent Manufacturing Co., Ltd.; PBS solution was acquired from Beijing Zhimaixing Biotechnology Co., Ltd.; cyclooxygenase-2 (COX-2) inhibitor screening assay kit and BeyGold™ solid black 96-well cell culture plates were purchased from Shanghai Beyotime Biotechnology Co., Ltd.; Human COX-2 enzyme (No. 0692731) was supplied by Cayman Chemical Company; and ultrafiltration tubes (10 and 30 kDa) were obtained from Millipore Corporation.

### 2.2 Sample preparation

#### 2.2.1 Sample and standards preparation

Medicinal slices of BS and ZGC were weighed at a 1:1 mass ratio following the classical prescription and transferred to a round-bottom flask. The mixture was immersed in distilled water (6-fold the total mass of herbal slices) for 30 min, followed by two rounds of heating under reflux, each lasting 1.5 h. All filtrates from each reflux extraction were combined after filtration, concentrated under reduced pressure, and lyophilized for subsequent experiments. The recovery yields of crude extracts and each ethanol gradient fraction separated from BS and ZGC were calculated using the following formula: Yield (%) = (dry weight of obtained fraction/weight of original raw herb) × 100%. Consistent with previously reported extraction profiles of Shaoyao Gancao Decoction via water reflux extraction, the extraction yield of crude BS extract was 18.62%, and that of crude ZGC extract was 16.37%.Approximately 1.00 g of the above lyophilized SGD powder was accurately weighed and transferred into a 10 mL volumetric flask. Methanol was added, and the sample was subjected to ultrasonic extraction (250 W, 40 kHz) for 30 min. After cooling to ambient temperature, methanol was supplemented to compensate for weight loss. The solution was fully mixed and centrifuged at 12,000 rpm for 5 min, and the supernatant was harvested. The supernatant was diluted to a final concentration of 0.1 mg/mL and filtered through a 0.22 μm microporous membrane to obtain the SGD test solution.

Reference standards of paeoniflorin, albiflorin, liquiritin and glycyrrhizic acid were accurately weighed and dissolved in 70% methanol to prepare a mixed standard working solution, with each single analyte maintained at a concentration of 10 μg/mL.

#### 2.2.2 Preparation of ethanol eluates at different concentrations for BS and ZGC

200 g of BS slices were subjected to reflux extraction twice using 1.6 L ultrapure water for 1 h per cycle. The combined filtrate was filtered, concentrated under reduced pressure, and lyophilized. The lyophilized BS powder was reconstituted with ultrapure water to prepare a stock solution of 0.2 g/mL, which was loaded onto pre-equilibrated D101 macroporous resin. The column was first washed with two bed volumes (BV) of 5% ethanol to deplete polysaccharides and polar impurities, followed by gradient elution with 3 BV each of 10%, 30%, 50%, and 70% ethanol. Each eluate was separately collected, concentrated, lyophilized, and preserved at 4 °C for subsequent analysis. The recovery yield of each BS gradient fraction was calculated as follows: 10% gradient (4.15%), 30% gradient (7.89%), 50% gradient (3.26%), 70% gradient (1.91%). The 30% gradient fraction exhibited the highest recovery among all BS eluted fractions.

For ZGC, 200 g raw material underwent identical water reflux extraction and D101 macroporous resin separation procedures as BS, with the only modification in impurity removal: 2 BV of 15% ethanol was adopted instead of 5% ethanol to wash off interfering polar components. Subsequent gradient elution was performed with 3 BV each of 15%, 30%, 50%, and 70% ethanol. All individual eluates were collected, concentrated under reduced pressure, lyophilized, and stored at 4 °C.The recovery yields of ZGC gradient fractions were measured as: 15% gradient (6.52%), 30% gradient (4.38%), 50% gradient (2.74%), 70% gradient (1.25%).

#### 2.2.3 Preparation of effective fractions of SGD

200 g BS was extracted by reflux according to 2.2.2., and the extract was then separated by pretreated D101 macroporous resin and eluted with 2 BV of 10% ethanol to remove polysaccharides and other impurities, followed by 6 BV of 40% ethanol, to obtain the 40% ethanol elution solution. The eluent solution was collected, concentrated under reduced pressure and freeze-dried to obtain the effective fractions of BS (rich in monoterpenoid glycosides).

200 g ZGC was also extracted according to 2.2.2., and then separated by pretreated D101 macroporous resin and eluted with 2 BV of 15% ethanol to remove impurities, followed by 6 BV of 40% and 80% ethanol. The eluent solutions were collected, concentrated under reduced pressure and freeze-dried to obtain the 40% and 80% ethanol eluates of ZGC (rich in flavonoids and triterpenoid/triterpenoid saponins, respectively).

### 2.3 UPLC-MS analysis conditions

UPLC-MS analysis was conducted on an Orbitrap Exploris 120 mass spectrometer equipped with a heated ESI source, which was coupled to a Vanquish™ UPLC system. A polarity switching method between ESI^−^ and ESI^+^ within a single run was enabled on mass spectrometer, and the parameters were set as follows: The spray voltage was 2.5 kV (−) and 3.5 kV (+); the UPLC-MS mode was applied with an Orbitrap resolution of 120,000 for full-MS and 15,000 for dd-MS^2^; the isolation window (*m/z*) was 2; the RF Lens% was 70; the sheath gas pressure was 45 Arb; the auxiliary gas pressure was 15 Arb; the sweep gas pressure was 0 Arb; the capillary temperature was 320 °C; the vaporizer temperature was 350 °C; the scanning range was *m/z* 100∼1000; and the normalized collision energies (NCE) were 15, 30 and 45 eV.

The HYPERSIL GOLD Vanquish C18 (2.1 mm × 100 mm, 1.9 µm) column was used in the system. The flow rate was 0.3 mL/min, the column temperature was 30 °C, and the injection volume was 1 µL. The 0.1% formate-water (A) and acetonitrile (B) were used as mobile phase, and the optimal gradient elution conditions in UPLC were: 0→35 min, 5%→71% B; 35→38 min, 71%→100% B.

### 2.4 UPLC-MS analyses of BS and ZGC (X matrix)

The quantitative matrix (X matrix) of chemical constituents in each ethanol eluate of BS and ZGC at different ethanol concentrations was analyzed using UPLC/Orbitrap Exploris 120 under the conditions specified in Section 2.2.3. Xcalibur software was employed to statistically analyze the changes in peak areas of common compounds in the different ethanol eluates of BS and ZGC, respectively (Chen et al., 2024).

### 2.5 *In vitro* COX-2 inhibition assays of BS and ZGC (Y matrix)

#### 2.5.1 Fluorescent COX-2 assay for ethanol fractions

*In vitro* COX-2 inhibitory activity of ethanol gradient fractions was determined with a commercial fluorescent COX-2 detection kit (Beyotime Biotechnology, Cat. BS-0089). All test fractions were predissolved in DMSO, and the final DMSO concentration in each reaction system was controlled at ≤0.1% (v/v) to eliminate solvent interference. Three independent biological replicates with two technical duplicates per concentration were set for all experimental groups. A black 96-well microplate was prepared, including blank control, 100% enzyme activity control, and serially diluted sample groups. After sample addition and mixing, the plate was incubated at 37 °C for 10 min, followed by sequential addition of 5 μL COX-2 probe and 5 μL COX-2 substrate working solution. The mixture was thoroughly mixed and incubated at 37 °C in the dark for 5 min, and fluorescence signals were recorded at Ex/Em = 560/590 nm. The relative fluorescence units (RFU) of blank wells, 100% activity control wells and sample wells were defined as RFU_blank_, RFU_100_% and RFU_sample_, respectively. The COX-2 inhibition rate was calculated by the following formula:

$$\text{Inhibition rate }\left(\%\right)=\left[\left({\text{RFU}}_{100}\%\;–\;{\text{RFU}}_{\text{sample}}\right)\;/\;\left({\text{RFU}}_{100}\%\;–\;{\text{RFU}}_{\text{blank}}\right)\right]\times 100\%$$

Celecoxib was used as the positive control under identical experimental conditions, and its IC_50_ value for fraction testing was calculated as 30.4 nM via nonlinear regression fitting.

#### 2.5.2 PGE_2_ EIA assay for purified monomeric compounds

To evaluate the COX-2 inhibitory capacity of pure monomeric compounds, a PGE_2_ enzyme immunoassay (EIA) was performed using a commercial PEG_2_ enzyme immunoassay (EIA) kit (Cayman Chemical, Cat. 514010)”. Briefly, each reaction tube contained 160 µL reaction buffer (0.1 M Tris-HCl, pH 8.0, supplemented with 5 mM EDTA and 2 mM phenol), 10 µL heme solution, 10 µL COX-2 enzyme and 10 µL monomer sample. For background control tubes, active COX-2 was replaced with denatured COX-2, and test samples were substituted with an equal volume of DMSO; 100% enzyme activity control tubes received equivalent DMSO instead of test inhibitors. All tubes were pre-incubated at 37 °C for 10 min, and 10 μL arachidonic acid (AA) was added to initiate the catalytic reaction. After incubation at 37 °C for 2 min, 30 μL stannous chloride solution was supplemented to terminate the reaction. The obtained prostanoid-containing solutions were diluted and transferred to 96-well plates pre-coated with mouse anti-rabbit monoclonal IgG antibody, along with prostaglandin-acetylcholinesterase conjugate tracer and EIA antiserum. The plates were incubated at 4 °C for 18 h with orbital shaking. Each well was washed five times to remove unbound reactants, and Ellman’s Reagent was added for color development under continuous shaking for 90 min to form yellow chromogenic products. The absorbance (OD) of each well was measured, and OD_blank_, OD_100_% and OD_sample_ represented the optical density of background control, full activity control and sample groups, respectively. The COX-2 inhibition rate was calculated as:

$$\text{Inhibition rate }\left(\%\right)=\left[\left({\text{OD}}_{100}\%\;–\;{\text{OD}}_{\text{sample}}\right)\;/\;\left({\text{OD}}_{100}\%\;–\;{\text{OD}}_{\text{blank}}\right)\right]\times 100\%$$

Celecoxib served as the positive control under consistent reaction conditions, and its IC_50_ value against COX-2 for monomer detection was determined to be 28.7 nM via nonlinear regression analysis.

### 2.6 Dose-effect (X-Y) matrix analyses of SGD

The peak areas of shared chemical constituents in each ethanol eluate of BS and ZGC were used as independent variables (X), while the COX-2 enzyme inhibition rate of each ethanol eluate was served as dependent variables (Y). An X-Y matrix dataset was established and imported into SIMCA 14.1 software for PLS analysis. Prior to modeling, missing peak signals of trace constituents were filled with zero values based on the component distribution of each herbal fraction dataset. Internal seven-fold cross-validation was adopted to select the optimal number of predictive and orthogonal components and calculate core statistical indicators R^2^X, R^2^Y and Q^2^. Permutation tests (n = 200) were further conducted to evaluate model overfitting risk. The corresponding summary of fit plots and permutation test diagrams are provided in Supplementary Material 1.

### 2.7 Screening potential COX-2-binding constituents by AUF-LC-MS

#### 2.7.1 Optimization of affinity ultrafiltration conditions

The SGD sample from Section 2.2.1 was dissolved in 5% DMSO to prepare a 20 mg/mL solution. COX-2 was dissolved in 10 mM PBS buffer (pH 6.8). A mixture of 100 μL SGD sample and 100 μL COX-2 (100, 200, or 400 U/mL) was incubated at 37 °C for 45 min. Replace COX-2 solution with PBS solution as the blank group, and add inactivated COX-2 (incubated at 100 °C for 10 min) as the control group. The reaction mixture was transferred to ultrafiltration membrane tubes (10 or 30 kDa) and centrifuged at 12,000 r/min (4 °C, 15 min). The membrane was washed three times with 300 μL PBS buffer. At this time, compounds which were not bound to COX-2 were washed out. Each sample was transferred to a new ultrafiltration tube, so as to reduce background interference due to non-specific binding with the device. Then, the COX-2 ligand complexes were treated with 300 μL 90% methanol solution and incubated at 4 °C for 10 min, followed by centrifugation at 12,000 rpm for 15 min. This procedure was repeated three times to gather the dissociated ligands. The 90% methanol ultrafiltrates from each sample were collected, evaporated to dryness using a vacuum centrifugal concentrator, and then reconstituted in 200 μL of 50% methanol. After vortex mixing, the solution was filtered through a 0.22 μm microporous membrane for UPLC-MS analysis. The binding degree (BD, %) was calculated as: BD = (A_2_−A_3_)/A_1_ × 100%, where A_1_, A_2_, and A_3_ represented peak areas of blank, experimental, and control groups, respectively.

#### 2.7.2 Screening of potential COX-2-binding constituents in SGD

Based on the optimal affinity ultrafiltration conditions determined in Section 2.7.1, the interaction between the aqueous extract of SGD and COX-2 enzyme was evaluated by calculating the BD values.

### 2.8 Data processing

Mass spectrometry raw data were acquired using Transfinder 5.1 and further analyzed with Xcalibur 3.0 software for peak alignment and extraction. A chemical constituent database of SGD was established by reviewing literature on the chemical compositions of BS and ZGC. Compound identification was performed by comparing retention times, molecular ion peaks, and fragment peaks with reference standards. The Compound Discoverer 3.2 online databases (mzCloud, mzVault, Chemspider, and MassList) were searched to match primary and secondary mass spectral fragments. Compounds with mass deviation <5 ppm and mzCloud match scores >60% were preliminarily identified. Further confirmation was achieved by analyzing diagnostic ions and fragmentation patterns reported in literature. All captured compounds were classified into two identification confidence grades: Level 1, definitive identification by matching retention time, precursor ion and MS/MS fragments with authentic reference standards; Level 2, tentative annotation only based on high-resolution fragment ions and published mass spectral data without standard comparison. Peaks judged as exogenous contaminants derived from solvents or plastic consumables were screened out and entirely removed from the compound identification table to avoid data interference.

The IC_50_ values were calculated using GraphPad Prism 8.0.2 software with a four-parameter logistic regression model, and all results were presented as mean ± standard deviation (mean ± SD). Potential bioactive components in the SGD aqueous extract were screened based on Variable Importance in Projection (VIP) values >1 and BD values >10%. Molecular weights, retention times (t_R_), and characteristic fragment ions acquired from MS/MS data using the Xcalibur workstation were combined with published literature and public databases to identify chemical constituents of the extract.

### 2.9 Molecular docking

Discovery Studio 2020 was utilized to perform molecular docking simulations between the 19 candidate bioactive compounds and RA-related target proteins (COX-2, PI3K, AKT, STAT3, JAK, EGFR, SRC, NFκB, HSP90AA1). The 2D structures of all small molecules were drawn via ChemOffice, followed by geometry optimization and energy minimization under the MMFF94 force field, then exported as mol2 format files. Human target crystal structures were retrieved from the RCSB PDB database (COX-2: 5GMM; PI3K: 4XZ4; AKT: 4EHZ; STAT3: 5AX3; JAK: 6AAH; EGFR: 3EQP; NFκB: 4G3G; SRC: 1Y57; HSP90AA1: 7LT0). Preprocessing steps for each protein included water molecule removal, deletion of irrelevant cofactors, addition of missing hydrogen atoms, and energy minimization using the CHARMm force field to obtain stable initial conformations. To verify the reliability of the docking workflow, re-docking validation was implemented: the co-crystallized ligand of COX-2 crystal structure was extracted, energy-optimized, and re-docked back into the original active pocket. The RMSD value between the re-docked optimal pose and the native crystal conformation was calculated as 1.12 Å (<2 Å), which confirmed the docking protocol was credible and reproducible. The binding pocket was defined as a 12 Å radius sphere centered on the geometric center of the co-crystallized ligand of each protein. The CDOCKER algorithm with CHARMm force field was adopted; the maximum energy minimization iteration was set to 5,000 steps, and CDOCKER interaction energy was taken as the primary scoring indicator to evaluate binding affinity. Celecoxib was used as the positive control for COX-2 docking comparison.

After docking calculation, all protein-ligand complex conformations were screened according to optimal binding scores. Residues within a 4 Å distance from each ligand were extracted to systematically analyze key interacting amino acids in the binding pocket, including residues forming stable hydrogen bonds and hydrophobic contacts with small molecules. All docking scoring data and key residue interaction information were summarized in Supplementary Table S2.

### 2.10 Molecular dynamics simulation

100 ns all-atom molecular dynamics (MD) simulations were performed on eight representative protein-ligand complexes, including COX-2-glycyrrhizic acid, COX-2-licoricesaponin H2, EGFR-licorice glycoside B, JAK-licorice glycoside B, STAT3-licorice glycoside B, PI3K-glycyrrhizic acid, PI3K-licorice glycoside B, and PI3K-licoricesaponin H2, using GROMACS 2022. The AMBER14SB force field was assigned to target proteins via the pdb2gmx module. Ligand structures were parameterized with the GAFF2 force field, and RESP partial atomic charges were generated using Sobtop 1.0. Each complex was solvated in a cubic TIP3P water box with a minimum 1.0 nm buffer distance from protein atoms to box boundaries. Na^+^ and Cl^−^ counterions were added to neutralize the system and maintain a physiological ionic strength of 0.15 M. Long-range electrostatic interactions were calculated via the PME algorithm with a 1.0 nm cutoff, and the LINCS algorithm was applied to constrain all hydrogen-containing covalent bonds. Before the production run, the system underwent multi-step energy minimization: 3,000 cycles of steepest descent followed by 2000 cycles of conjugate gradient optimization. Sequential NVT (310 K, Nosé-Hoover thermostat) and NPT (1 bar, Parrinello–Rahman barostat) equilibration simulations were each conducted for 100 ps. The 100 ns production MD trajectory was run with a 2 fs integration timestep. Standard GROMACS analysis tools were used to extract five core trajectory metrics for all complexes: backbone RMSD to evaluate global conformational stability, residue RMSF to reflect local amino acid flexibility, radius of gyration (Rg) to assess overall protein compactness, solvent accessible surface area (SASA) to monitor solvent exposure changes, and time-resolved intermolecular hydrogen bond counts to characterize persistent polar interactions between ligands and binding pockets. Based on RMSF profiles, residues with low fluctuation values within the ligand-binding cavity were qualitatively identified as critical residues that stabilize ligand binding. All time-dependent trajectory curves for the eight multi-target complexes are compiled and displayed in Supplementary Material 1; only representative MD results related to the core target COX-2 are presented in the main text to avoid redundant multi-pathway data discussion. Limitation note: Owing to limited computational resources, only a single independent 100 ns trajectory was generated for each complex. Quantitative binding free energy decomposition via MM/GBSA or MM/PBSA was not performed in the present work. Parallel repeated MD simulations and systematic MM/PBSA free energy calculations will be implemented in our follow-up computational research.

### 2.11 *In vitro* bioassay verification

#### 2.11.1 COX-2 inhibition assays of compounds and effective fractions of BS and ZGC

The COX-2 inhibitory activities of compounds and effective fractions of BS and ZGC were tested by referring to Section 2.5.

#### 2.11.2 NO inhibition of SGD and SGF on LPS-stimulated RAW264.7 cells

The RAW 264.7 cells (Procell Life Science and Technology Co. Ltd.) were cultured in Dulbecco’s modified Eagle’s medium (DMEM) with 10% fetal bovine serum (FBS), 100 U/mL penicillin and 100 U/mL streptomycins at 37 °C in a humidified atmosphere with 5% CO_2_. Cell viability was evaluated by MTT. The assay was performed by culturing the cells (1 × 10^5^ cells/mL) in 96-well plates for 12 h, followed by treatment with the tested SGD and SGF in different concentrations for 24 h. After adding 20 μL of MTT (5 mg/mL) to each well, the samples were incubated for 4 h. Removing the liquid, 150 μL DMSO was added to each well and the plate was shaken on an oscillator for 10 min to completely dissolve the formazan crystals. And the absorbance at 490 nm was measured by using a microplate reader.

NO production was determined by measuring the nitrite concentration in the culture supernatant using Griess reagent. The cells (2 × 10^6^ per well) were seeded in 12-well plates for 12 h and then treated with SGD and SGF at 100 and 200 μg/mL for 1 h. Dexamethasone (Sigma) was used as the positive control. 2 μg/mL LPS was added and cultured for 24 h. 50 μL of the supernatant from incubates was mixed with an equal volume of Griess reagent (1% sulfanilamide in 5% phosphoric acid; 0.1% naphthyl ethylenediamine dihydrochloride in distilled water) at 37 °C. After 10 min, the absorbance was measured at 540 nm. The amount of NO release was calculated.

## 3 Results and discussion

### 3.1 COX-2 inhibitory effect of SGD

The inhibitory activity of SGD toward COX-2 was firstly evaluated using fluorescent COX-2 detection kit. Celecoxib was used as the positive control, which was a selective COX-2 inhibitor. As shown in Figure 1A, the aqueous extract of SGD exhibited significant inhibitory activity against COX-2, with an IC_50_ value of 62.5 μg/mL, the dose–response curve of celecoxib (IC_50_ = 30.4 nM) for this assay is provided in Supplementary Material 1.

**FIGURE 1:**
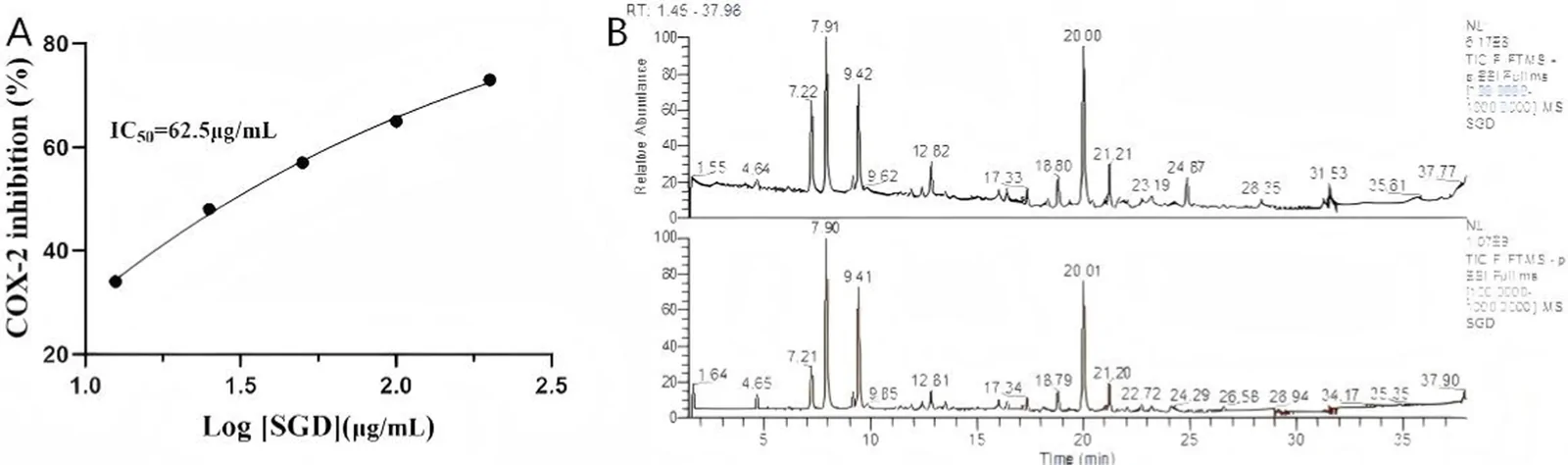
COX-2 inhibitory activity **(A)** and total ion chromatograms **(B)** of the aqueous extract of SGD. **(A)** Dose-response curve for COX-2 inhibition by the aqueous extract of SGD; IC_50_ = 62.5 μg/mL. Celecoxib positive control data (IC_50_ = 30.4 nM) are shown in Supplementary Material 1. **(B)** Representative total ion chromatograms (TICs) of the aqueous extract of SGD acquired under ESI^+^ and ESI^−^ modes. Dose-response curves represent global four-parameter logistic regression fits for IC_50_ computation; individual replicate raw data are provided in Supplementary Material 3.

### 3.2 Component identification of SGD

The chemical constituents of SGD were analyzed using UPLC/Orbitrap Exploris 120 mass spectrometer. The identification was performed based on relative molecular mass, mass spectral fragmentation patterns, comparison with reference standards, literature reports, an in-house database, and non-targeted online databases (including mzCloud, mzVault, ChemSpider, and MassList). A total of 153 endogenous constituents were detected. Four compounds marked with asterisks (*) were verified against authentic reference standards, whereas the remaining 149 compounds were tentatively characterized without standard validation (Supplementary Material 1). The total ion chromatograms (TIC) of SGD in positive and negative ion modes were shown in Figure 1B.

### 3.3 Establishment of the dose-effect (X-Y) matrix of SGD

#### 3.3.1 COX-2 inhibitory effects of different ethanol elutes from BS and ZGC (Y matrix)

As shown in Figures 2A–H, the ethanol eluates obtained from BS using D101 macroporous adsorption resin with 10%, 30%, 50%, and 70% ethanol aqueous all exhibited significant inhibitory effects on COX-2, with IC_50_ values ranging from 27.63 to 121.3 μg/mL. Among them, the 30% ethanol eluate demonstrated the strongest inhibitory effect, with an IC_50_ value of 27.63 μg/mL. The ethanol eluates of ZGC obtained through macroporous resin using 15%, 30%, 50% and 70% ethanol aqueous all exhibited certain COX-2 inhibitory effects, with IC_50_ values ranging from 101.3 to 234.6 μg/mL. Among them, the 70% ethanol eluate showed the best inhibitory effect, with an IC_50_ value of 101.3 μg/mL. According to the above, it can be seen that high polar elution fractions of BS had better COX-2 inhibitory activity, especially for 30% and 50% ethanol elutes, which provided an important clue to search for the effective substances as well as effective fractions of SGD. Notably, the refined 40% ethanol eluate of BS prepared by targeted enrichment exhibited even stronger COX-2 inhibitory activity (IC_50_ = 23.8 μg/mL) compared with all serial gradient fractions mentioned above.

**FIGURE 2:**
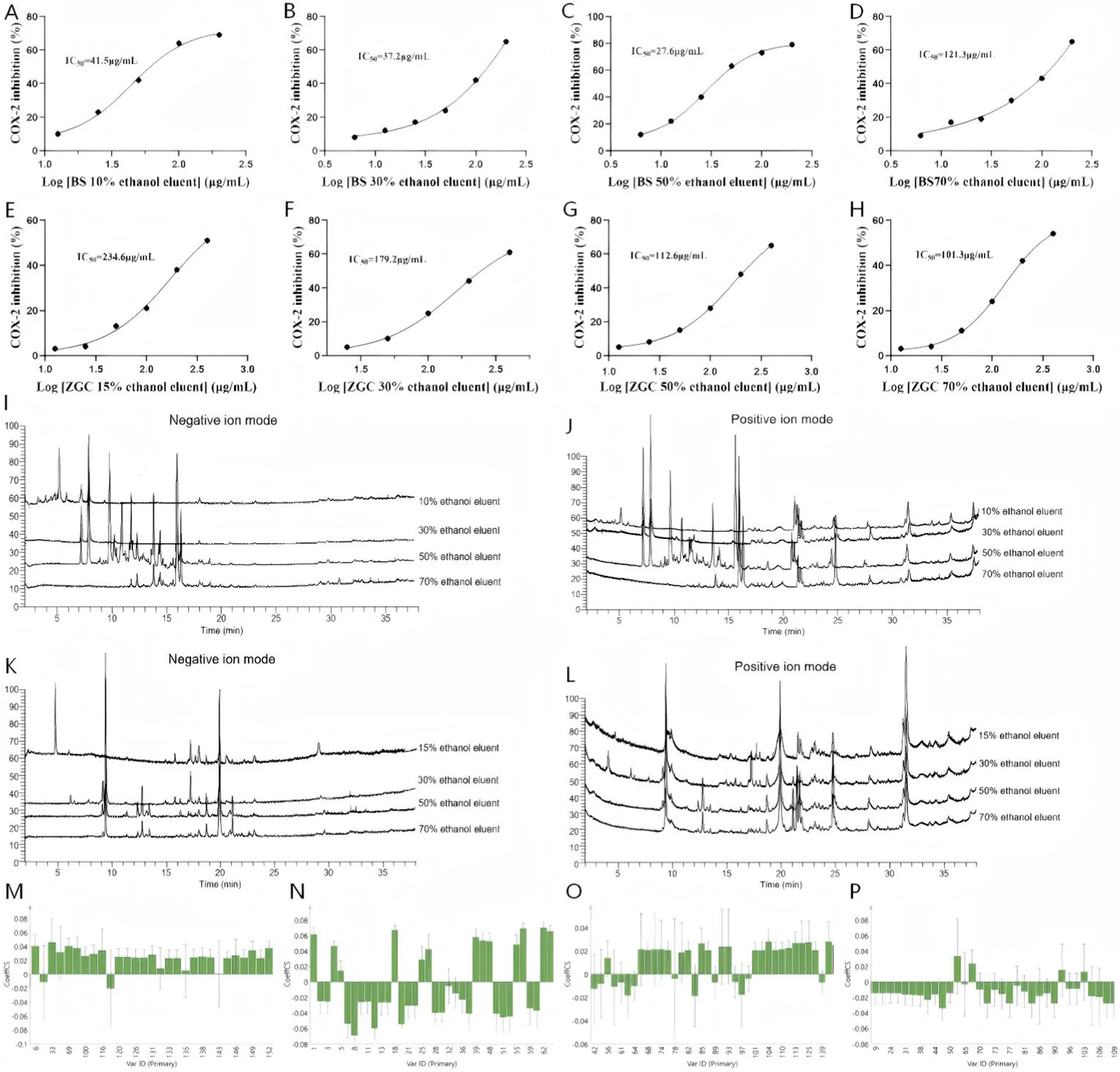
PLS model of SGD. **(A–H)** The COX-2 inhibitory effects of different ethanol eluates of BS and ZGC ( $\bar{x}\pm s$ ,n = 3); Celecoxib positive control data (IC_50_ = 30.4 nM) are shown in Supplementary Material 1. **(I–L)** Total ion chromatogram of different ethanol eluates of BS and ZGC; **(M–P)** Coefficient diagram of partial components in BS and ZGC under positive and negative ion modes. Dose-response curves represent global four-parameter logistic regression fits for IC_50_ computation; individual replicate raw data are provided in Supplementary Material 3.

#### 3.3.2 Analysis of components in different ethanol eluates from BS and ZGC (X matrix)

The different ethanol elutes of BS and ZGC (Figures 2I–L) were analyzed by UPLC/Orbitrap Exploris 120. The peak areas extracted by Xcalibur software can reflect the changes in the contents of chemical components in the different ethanol eluates. The quantitative matrix of SGD was established based on the peak area variations of components in different BS and ZGC eluates, analyzed in both positive and negative ion modes. During construction of the X matrix, peak intensities of individual analytes differed substantially across ethanol gradient fractions. For trace components undetectable in certain eluates (yielding blank chromatographic peaks), missing values were replaced with zero values in accordance with the compositional distribution trends observed from macroporous resin separation. This standard metabolomics preprocessing strategy was adopted to avoid bias to subsequent PLS regression modeling.

#### 3.3.3 Correlation analysis of the dose-effect matrix for SGD

The PLS model was constructed based on eight ethanol gradient elution samples, including four concentration gradients of BS and four of ZGC. A total of 153 mass spectrometric chromatographic peaks were defined as independent variables (X matrix), and the corresponding COX-2 inhibitory rates were set as the dependent variable (Y matrix). Internal seven-fold cross-validation was implemented to select the optimal number of model components and calculate key statistical parameters R^2^X, R^2^Y, and Q^2^. Permutation tests (n = 200) were further conducted to assess overfitting risk. Summary of fit plots obtained from seven-fold cross-validation guided the selection of optimal components. The PLS models of ZGC (negative/positive ion modes) adopted one predictive component and three orthogonal components, while BS models (negative/positive ion modes) used one predictive component plus two orthogonal components. Subsequent 200-time permutation tests confirmed the absence of overfitting in all four optimized models (Supplementary Material 1). In positive ion mode, the PLS model yielded R^2^X = 0.633, R^2^Y = 0.994, and Q^2^ = 0.966, representing the proportion of variance in X explained by the model, the explained variance of Y, and the predictive capacity, respectively.

Similarly, in negative ion mode, the PLS model results were R^2^X = 0.816, R^2^Y = 0.923, and Q^2^ = 0.951, demonstrating good predictive capability for the dataset in both positive and negative modes. The coefficient plots of the PLS model illustrated the correlation strength between the Y-matrix and each X-variable. The partial results were selected for analysis as shown in Figures 2M–P, the coefficient magnitudes represented the changes in Y variables when X variables shifted from 0 to 1. The dependent variable Y was collectively influenced by multiple independent variables, suggesting that the COX-2 inhibition of BS resulted from the individual activities of various constituent compounds rather than a single compound.

In positive ion mode, the PLS model analyses of ZGC yielded R^2^X = 0.564, R^2^Y = 0.982, and Q^2^ = 0.809, indicating that the model explained 56.4% of the variance in the X matrix, 98.2% of samples fitted the regression model, with a predictive ability of 80.9%. In negative ion mode, the PLS model results were R^2^X = 0.795, R^2^Y = 0.955, and Q^2^ = 0.905, demonstrating good predictive performance for the dataset in both positive and negative modes. As shown in the PLS coefficient plots (Figures 2M–P), the dependent variable Y was collectively influenced by multiple independent variables X, indicating that the COX-2 inhibition of ZGC was caused by the effects of multiple complex compounds.

In PLS analysis, the Variable Importance in Projection (VIP) scores reflected the relative contribution of each independent variable (X) in explaining the variance of the dependent variable (Y). A higher VIP value indicated stronger explanatory capability of variable X. Based on the PLS model, compounds with VIP >1 were screened. In BS, 22 and 19 compounds were identified in positive and negative ion modes, respectively, while in ZGC, 23 and 39 compounds were detected in these respective ion modes, respectively. Cross-validation and permutation test results verified that the established PLS regression models for BS and ZGC exhibited no obvious overfitting.

### 3.4 Screening of potential COX-2-binding constituents in SGD by affinity ultrafiltration mass spectrometry

#### 3.4.1 Optimization of affinity ultrafiltration conditions

The optimal conditions for affinity ultrafiltration were systematically investigated by evaluating the effects of different COX-2 enzyme concentrations and ultrafiltration membrane molecular weight cut-offs (MWCO) on the binding efficiency between 20 mg/mL SGD and COX-2. Principal Component Analysis (PCA) results (Figures 3A,B) revealed that increasing COX-2 concentrations enhanced the metabolomic profile differences between the COX-2 reaction group (*Spl400U)* and the inactivated COX-2 control group (*Blk400U*), indicating greater ligand-enzyme binding. Consequently, 400 U/mL COX-2 was selected as the optimal enzyme concentration. Comparative evaluation of 10 kDa and 30 kDa ultrafiltration membranes demonstrated that both could effectively differentiate the COX-2 reaction group from the control group. However, the 30 kDa membrane showed better retention of COX-2 inhibitor complexes based on dispersion patterns in the PCA plots. Therefore, the combination of 400 U/mL COX-2 and 30 kDa ultrafiltration membrane was established as the optimal affinity ultrafiltration condition for subsequent experiments.

**FIGURE 3:**
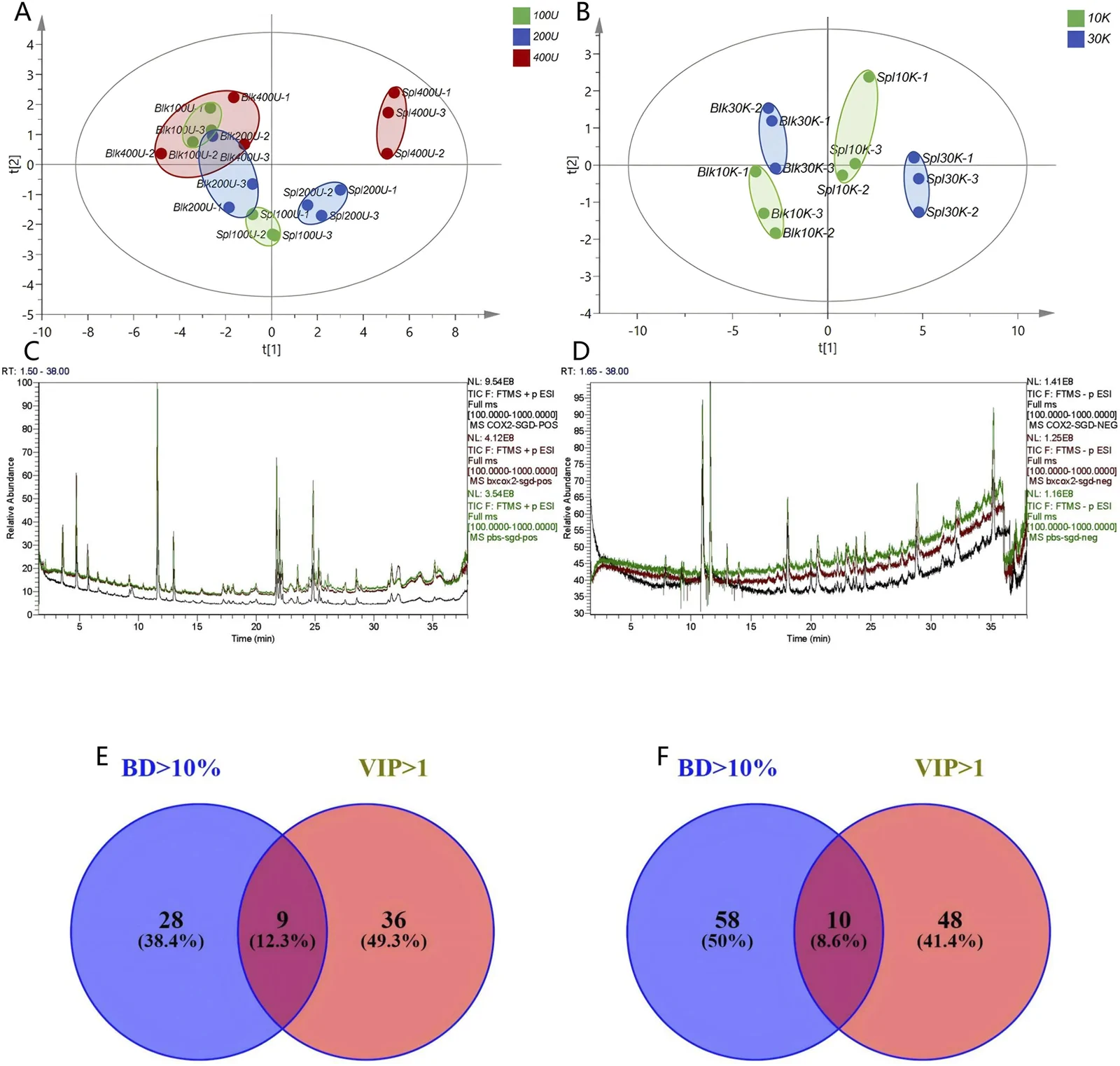
Screening results of potential COX-2-binding constituents in SGD by AUF-LC-MS. **(A,B)** Optimization of affinity ultrafiltration conditions. *Spl 100U* and *Blk 100U* represented the reactions of 100 U/mL COX-2 and inactivated COX-2 with the sample, respectively; reactions at 200 U/mL and 400 U/mL were denoted similarly as *Spl 200U* and *Blk 200U*, and *Spl 400U* and *Blk 400U,* respectively; *Spl 10K* and *Blk 10K* represented the reactions of COX-2 and inactivated COX-2 with the sample in 10 kDa affinity ultrafiltration tubes, respectively; reactions in 30 kDa affinity ultrafiltration tubes were denoted similarly as *Spl 30K* and *Blk 30K*. All groups were analyzed in triplicate. **(C,D)** Total ion chromatograms of the experimental group (black), denatured control group (red), and blank control group (green). **(E,F)** Screening results of potential COX-2-binding constituents in SGD by AUF-LC-MS in positive and negative ion modes.

#### 3.4.2 Screening of potential COX-2-binding constituents in SGD by AUF-LC-MS

The AUF-LC-MS method was employed to screen for compounds in SGD with affinity to COX-2 enzyme. The total ion chromatograms in positive and negative ion modes are shown in Figures 3C,D. The peak areas of compounds from each group were extracted, and the BD values were calculated. Potential components with BD >10% were systematically summarized. The results revealed that a total of 105 compounds exhibited BD values > 10% across both ionization modes. Among these, BS contributed 19 and 37 compounds in positive and negative ion modes, respectively, while ZGC yielded 18 and 31 compounds in the respective modes.

### 3.5 Screening results of potential COX-2-binding constituents

The PLS model prediction results (VIP>1) and AUF-LC-MS screening of potential COX-2-binding constituents (BD>10%) were mutually verified using Venn diagram analysis. As shown in Figures 3E,F and Supplementary Figure S1(Supplementary Material 1), and Table 1, a total of 19 active components were identified across both positive and negative ion modes, including two monoterpenoid glycosides, eight triterpenoids and triterpenoid saponins, eight flavonoids and one fatty acid. These compounds were sorted by BD values in Table 1, with the top five being paeoniflorin, glycyrrhizic acid, licorice glycoside B, licoricesaponin H2 and licoricesaponin G2. Among them, two compounds were from BS, while 17 compounds from ZGC.

**TABLE 1:** Potential COX-2-binding constituents from SGD.

NO.	Names	Structural types	BD (%)	VIP	Resource
28	Paeoniflorin*	Monoterpenoid glycosides	79	1.14	BS
108	Glycyrrhizic acid*	Triterpenoid saponins	78	1.23	ZGC
75	Licorice glycoside B	Chalcone glycosides	72	1.24	ZGC
115	Licoricesaponin H2	Triterpenoid saponins	71	1.07	ZGC
85	Licoricesaponin G2	Triterpenoid saponins	66	1.21	ZGC
112	Glycyrrhetinic acid*	Triterpenoids	62	1.13	ZGC
110	Uralsaponin B	Triterpenoid saponins	61	1.23	ZGC
99	Glabrolide	Triterpenoids	56	1.13	ZGC
113	3-Oxoglycyrrhetinic acid	Triterpenoids	55	1.13	ZGC
38	Violanthin	Flavonoid glycosides	53	1.18	ZGC
32	Albiflorin*	Monoterpenoid glycosides	48	1.25	BS
47	Liquiritin apioside	Dihydroflavonoid glycosides	47	1.10	ZGC
67	Liquiritin	Dihydroflavonoid glycosides	37	1.25	ZGC
77	Liquiritigenin	Dihydroflavonoids	34	1.14	ZGC
34	Engeletin	Dihydroflavonol glycosides	28	1.20	ZGC
102	Isoglabrolide	Triterpenoids	27	1.08	ZGC
69	Ononin	Isoflavonol glycosides	26	1.05	ZGC
70	Isoliquiritin	Chalcone glycosides	22	1.17	ZGC
83	Jasmonic acid	Fatty acids	16	1.04	BS/ZGC

**BD**, binding degree, calculated as (BD = (A_2_−A_3_)/A_1_ × 100%) from affinity-ultrafiltration LC-MS; **VIP**, Variable Importance in Projection derived from partial-least-squares (PLS) regression modelling.

Compounds marked with *were identified by authentic reference standards and validated for COX-2 inhibitory activity by *in vitro* enzyme assay.

### 3.6 Molecular docking verification

A total of 19 candidate potential COX-2-binding constituents screened from SGD (including paeoniflorin, glycyrrhizic acid, licorice glycoside B, *etc.*, Table 1) were subjected to molecular docking against COX-2 (PDB ID: 5GMM). Compounds with a CDOCKER interaction energy ≤ −5.0 kJ/mol were considered to possess favorable binding affinity toward the target protein. Docking results (Supplementary Material 1) revealed that all 19 compounds exhibited good binding affinity for COX-2. Among these candidates, triterpenoid saponins displayed stronger binding capacity. Licoricesaponin H2, licoricesaponin G2, uralsaponin B and glycyrrhetinic acid yielded CDOCKER interaction energies of −78.16, −71.84, −68.65 and −64.08 kJ/mol, respectively.

The 19 active compounds were further docked to key target proteins associated with rheumatoid arthritis signaling pathways. As summarized in the docking affinity heatmap (Figure 4), most compounds showed strong binding affinities to PI3K, STAT3, JAK, SRC,NFκB, EGFR and AKT. For the PI3K target, licoricesaponin H2, glycyrrhizic acid, uralsaponin B and licoricesaponin G2 exhibited interaction energies of −86.29, −85.50, −83.87 and −82.25 kJ/mol, respectively. Licorice glycoside B and violanthin displayed high affinity to STAT3, with energies of −85.34 and −84.11 kJ/mol. Licorice glycoside B and liquiritin apioside obtained favorable binding energies of −81.38 and −70.17 kJ/mol against JAK, while violanthin and licorice glycoside B reached −77.18 and −76.25 kJ/mol for EGFR. The detailed 2D and 3D binding modes of representative compounds (licoricesaponin H2, glycyrrhizic acid, and licorice glycoside B) with their respective targets are illustrated in Figure 5.

**FIGURE 4:**
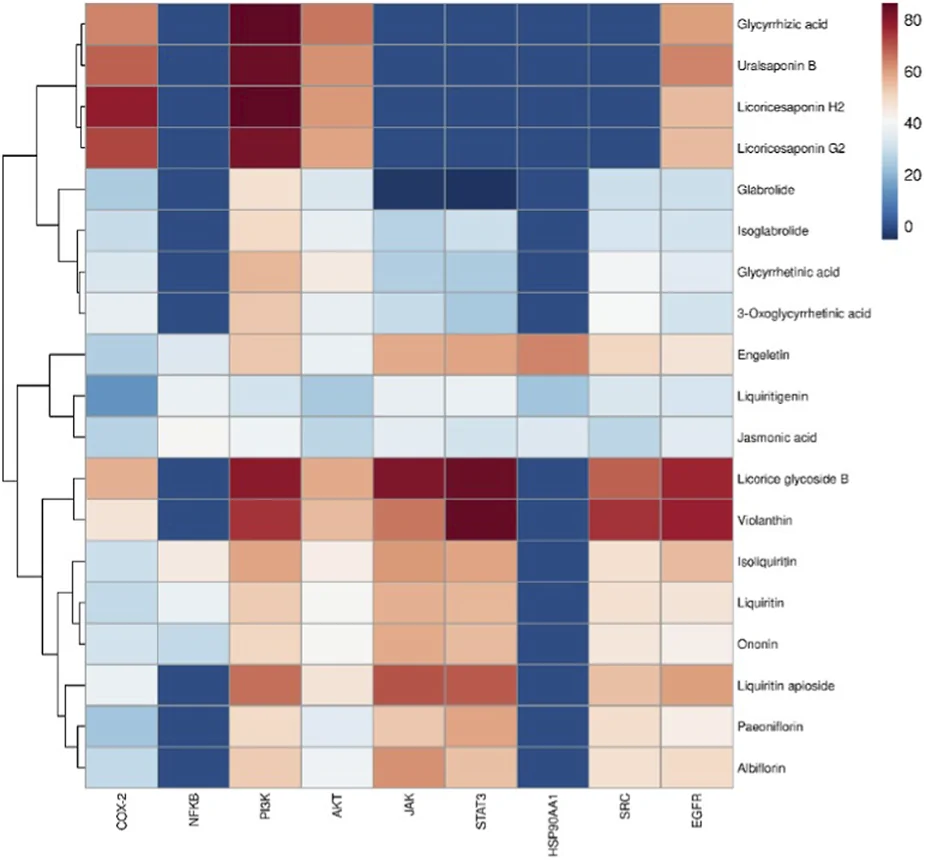
Heatmap of molecular docking. The colour gradient scale (0-20-40-60-80) represents absolute values of CDOCKER interaction energy (kJ/mol). Higher numerical values indicate stronger predicted protein-ligand binding affinity. Heatmap values were directly exported from Discovery Studio 2020 molecular-docking outputs.

**FIGURE 5:**
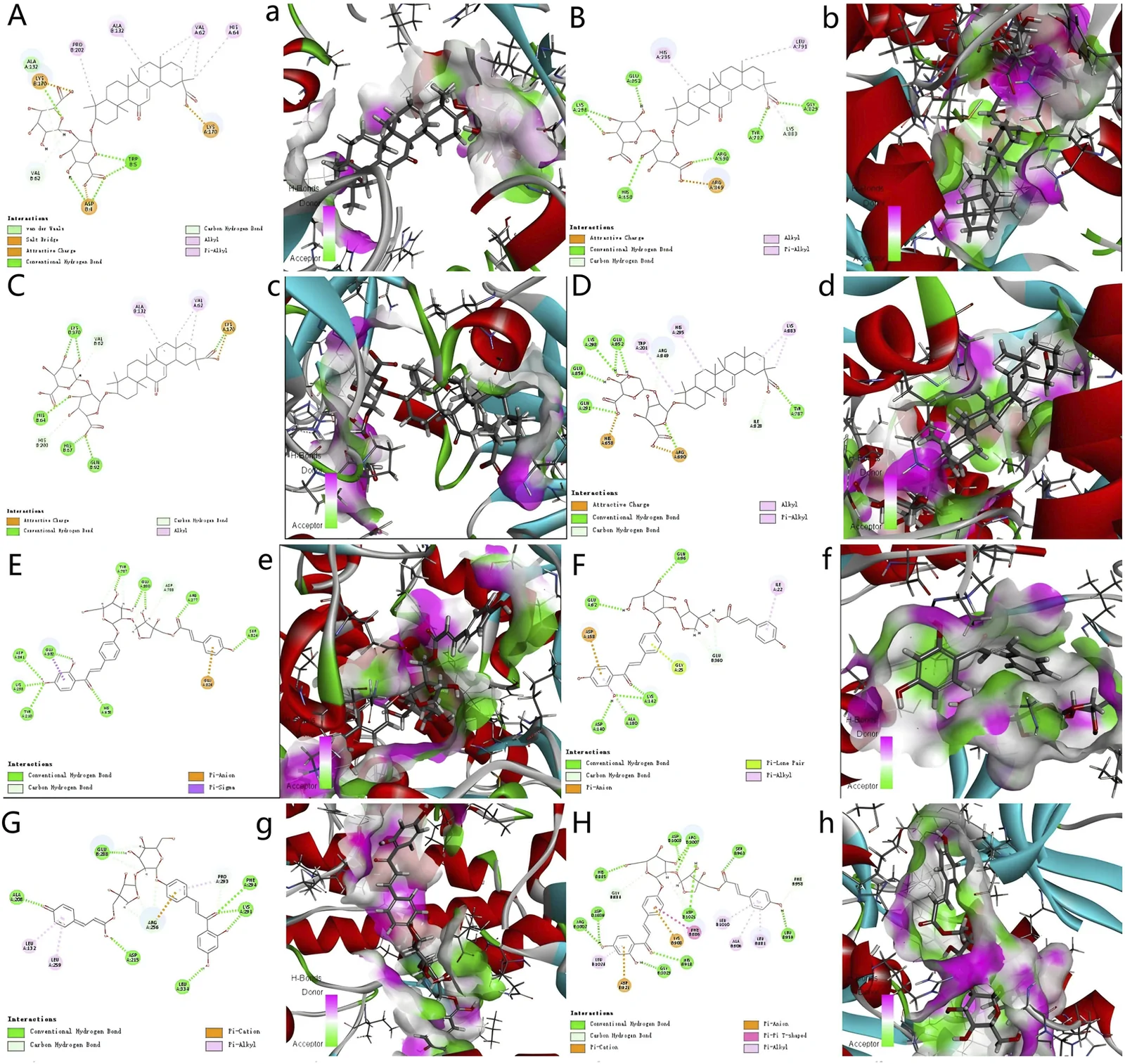
Molecular docking simulations between typical bioactive compounds and target proteins (COX-2, PI3K, STAT3, EGFR, JAK). **(A,a,B,b)** Docking results of licoricesaponin H2 bound to COX-2 and PI3K, respectively; **(C,c,D,d)** Docking conformations of glycyrrhizic acid complexed with COX-2 and PI3K, respectively; **(E,e,F,f,G,g,H,h)** Binding modes of licorice glycoside B interacting with PI3K, STAT3, EGFR and JAK, respectively.

Collectively, these docking results indicated that SGD treats rheumatoid arthritis via a multi-component, multi-target and multi-pathway mode of action. The potential underlying mechanisms are closely related to the PI3K-Akt and JAK-STAT signaling pathways, as well as inflammation-associated signaling cascades.

### 3.7 Molecular dynamics simulation

In this work, 100 ns molecular dynamics (MD) simulations were carried out to assess the structural stability of the constructed protein–ligand complexes (Mark and Nilsson, 2001). Root-mean-square deviation (RMSD) values calculated based on protein backbone atoms were employed to track global conformational variations of each system throughout the simulation. For COX-2-associated systems, the COX-2-glycyrrhizic acid complex reached equilibrium after 20 ns, with RMSD values steadily fluctuating around 2.3 Å. The COX-2-licoricesaponin H2 complex achieved structural equilibrium at 40 ns, accompanied by minor RMSD fluctuations centered at 2.0 Å. The EGFR–licorice glycoside B complex required a longer equilibration phase and stabilized after 70 ns, maintaining steady fluctuations near 2.6 Å. The JAK–licorice glycoside B complex remained stable from 50 ns to 90 ns; although a slight upward trend in RMSD was observed, the overall fluctuation was kept below 8.0 Å. All three PI3K-related complexes rapidly reached equilibrium within the initial 5–10 ns, with stable RMSD fluctuations ranging from 3.2 Å to 3.7 Å. The STAT3-licorice glycoside B complex retained a relatively stable conformation across most simulation time, and its fluctuation amplitude was consistently less than 2.4 Å. Overall, all ligand-protein systems displayed well-converged RMSD profiles, revealing that these candidate compounds could bind to corresponding targets and form structurally stable complexes.

Hydrogen bonding constitutes a vital noncovalent interaction sustaining ligand–protein binding stability. Accordingly, the dynamic variation in hydrogen bond counts throughout the full 100 ns simulation trajectory was statistically analyzed (Supplementary Material 1). The results revealed sustained hydrogen bond generation in all protein–ligand complexes over the simulation period, with average hydrogen bond numbers stably maintained at 4–7 for each system. This observation confirmed durable, effective intermolecular interactions between the licorice-derived candidate constituents and target proteins. Combined with backbone RMSD convergence analysis, the 100 ns molecular dynamics (MD) simulations verified that glycyrrhizic acid, licoricesaponin H2 and licorice glycoside B could stably associate with COX-2, EGFR, JAK, PI3K and STAT3. Further statistical profiling of residue contact interactions illustrated that these compounds formed consistent hydrogen bonds and hydrophobic contacts with conserved amino acid residues within target active pockets, which cooperatively stabilized the overall protein–ligand binding conformation throughout the simulation.

### 3.8 *In vitro* verification

#### 3.8.1 COX-2 inhibitory effects of compounds and effective fractions of BS and ZGC

The results of *in vitro* enzyme activity experiments showed that paeoniflorin, albiflorin, glycyrrhizic acid and glycyrrhetinic acid all had good inhibitory effects on COX-2, with IC_50_ values of 23.8, 53.2, 54.1 and 58.9 μM. Celecoxib was set as the positive control under identical EIA conditions, with an IC_50_ of 28.7 nM; its corresponding dose–response curve is displayed in Supplementary Material 1. These inhibitory data were consistent with the affinity ultrafiltration screening results, as shown in Figure 6.

**FIGURE 6:**
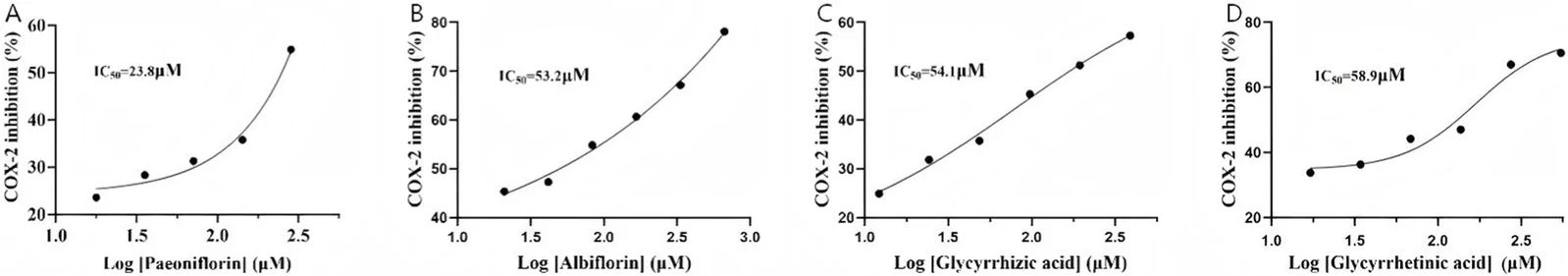
Dose-response curves illustrating the COX-2 inhibitory effects of individual bioactive compounds ( $\bar{x}\pm s$ ,n = 3). All inhibitory curves were determined via PGE_2_ EIA assay; the celecoxib positive control yielded IC_50_ = 28.7 nM under identical reaction conditions (Supplementary Material 1). Dose-response curves represent global four-parameter logistic regression fits for IC_50_ computation; individual replicate raw data are provided in Supplementary Material 3.

Based on the above findings, we also evaluated the *in vitro* COX-2 inhibition of effective fractions of BS and ZGC, including 40% ethanol eluate of BS (rich in monoterpenoid glycosides), 40% and 80% ethanol eluates of ZGC (rich in flavonoid glycosides and triterpenoid/triterpenoid saponins, respectively). As shown in Figure 7, the IC_50_ value of the 40% ethanol eluate of BS was 23.8 μg/mL, and the COX-2 inhibition effect of this fraction was significantly better than that of all the other ethanol eluates of both BS and ZGC; The IC_50_ values of 40% and 80% ethanol eluates were 114.6 and 98.3 μg/mL, respectively, and 80% ethanol eluate showed the best COX-2 inhibitory effect in all the different ethanol eluates of ZGC.

**FIGURE 7:**
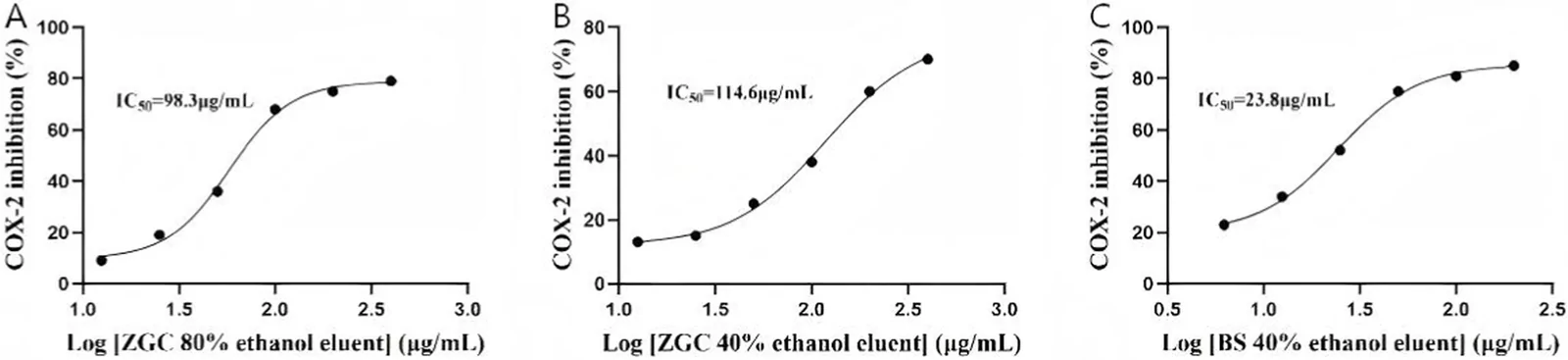
COX-2 inhibitory activity of BS and ZGC fractions ( $\bar{x}\pm s$ ,n = 3). Celecoxib positive control data (IC_50_ = 30.4 nM) are shown in Supplementary Material 1. Dose-response curves represent global four-parameter logistic regression fits for IC_50_ computation; individual replicate raw data are provided in Supplementary Material 3.

#### 3.8.2 Comparison of NO inhibition of SGD and SGF on LPS-stimulated RAW264.7 cells

According to the 1:1 ratio of two herbal medicines in the formula, mix the 40% ethanol fraction of BS and 40% and 80% fractions of ZGC to obtain the effective fractions of SGD (SGF). *In vitro* NO inhibition effects of both SGD and SGF were evaluated on LPS-stimulated RAW264.7 cells.

Compared to the blank control group, both SGD and SGF exhibited negligible cytotoxicity towards RAW264.7 cells at concentrations below 200 μg/mL (Figures 8A,B). The inhibitory effects of SGD and SGF at 100 and 200 μg/mL on LPS-induced NO production in RAW264.7 cells were further investigated. Both concentrations of SGD and SGF significantly reduced the LPS-induced increase in NO levels (*P* < 0.01), with a concentration dependent manner, and SGF exhibited greater NO inhibition compared to SGD. These findings suggested that the effective fraction of SGD possessed stronger anti-inflammatory activity and it was worth further studying the efficacy and mechanism of SGF. These results provide a foundation for developing novel combinatorial drugs based on the effective fractions of individual herbs, offering a promising approach to advance the high-quality development of Chinese formulas.

**FIGURE 8:**
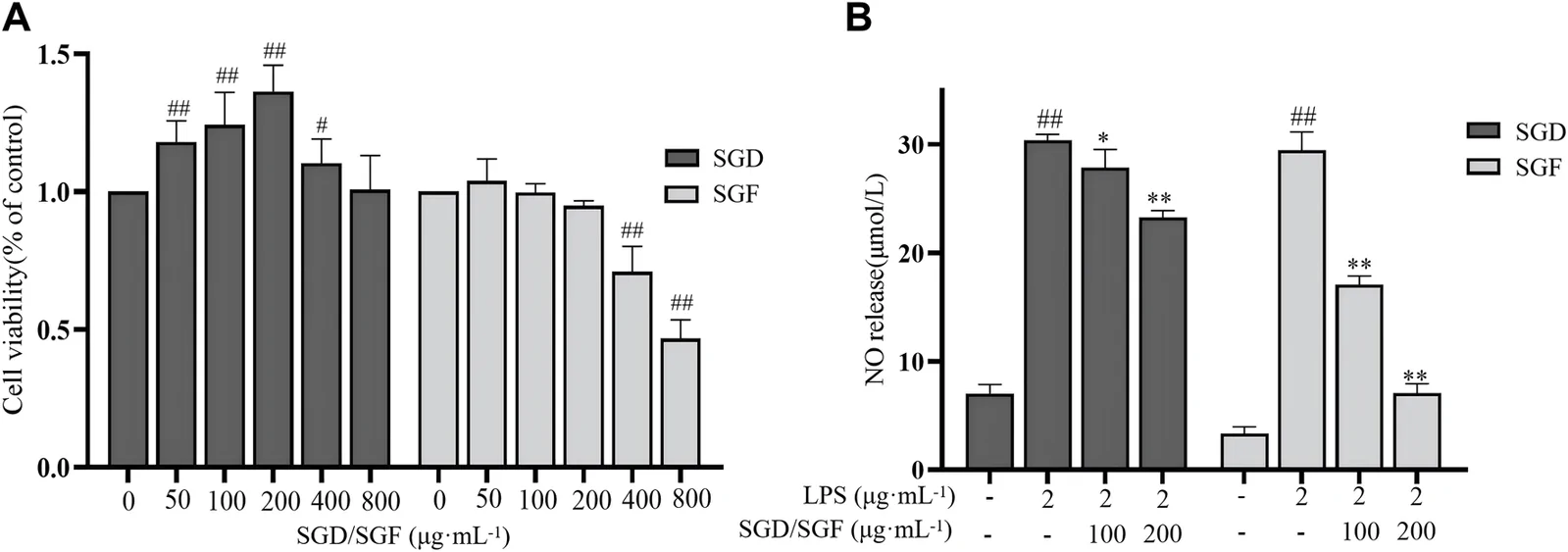
Effects of SGD and its effective fraction SGF on cell viability **(A)** and NO production **(B)** in RAW264.7 cells ( $\bar{x}\pm s$ , n = 3). **(A)** #*p* < 0.05, ##*p* < 0.01 versus control group. **(B)** ##*p* < 0.01 versus control group; *p < 0.05, **p < 0.01 versus LPS-stimulated control group, one-way ANOVA followed by Dunnett’s *post hoc* test.

### 3.9 Discussion

The present NO inhibition assay demonstrated that the reconstituted active fraction SGF exerted stronger anti-inflammatory effects than the crude SGD extract in LPS-stimulated RAW264.7 macrophages. Accumulating pharmacological studies have verified the anti-rheumatoid arthritis activity of SGD. Accumulating pharmacological studies have verified the anti-rheumatoid arthritis activity of SGD. A previously published study (Zhu et al., 2024) demonstrated that SGD treatment significantly downregulated the expression of COX-2, IL-1β and TNF-α in LPS-stimulated RAW264.7 macrophages, preliminarily indicating a potential correlation between the anti-inflammatory efficacy of SGD and COX-2 regulation. Nevertheless, such *in vitro* cellular evidence alone cannot fully confirm that the complete SGD formula exerts anti-RA therapeutic effects specifically via COX-2-dependent signaling pathways. It should be clarified that the present study mainly focuses on screening single chemical constituents with binding capacity to COX-2 and verifying their isolated *in vitro* COX-2 enzyme inhibitory activity, rather than systematically validating the overall COX-2-targeted anti-RA mechanism of the whole decoction through standardized *in vivo* arthritis models. Further systematic pharmacological validation in CIA rats is still required to solidify this mechanistic inference.

## 4 Conclusion

In the present study, a comprehensive and systematic medicinal chemistry research strategy was established by integrating AUF-UPLC-MS targeted screening, fraction-activity correlation analysis, *in vitro* enzymatic activity verification, and computational molecular simulation, enabling efficient mining and structural characterization of potential COX-2-binding constituents from the complex chemical matrix of SGD. The PLS fraction-activity models demonstrated good predictive validity (Q^2^ > 0.8) for both BS and ZGC, supporting the reliability of the spectrum-effect correlation approach. A total of 19 constituents with COX-2 binding capacity were screened, among which four monomers were verified to possess definite *in vitro* COX-2 inhibitory activity, covering multiple chemical skeletons including monoterpenoid glycosides, triterpenoids, saponins. Statistical constituent source analysis showed that two bioactive compounds were derived from BS, while 17 constituents originated from ZGC. This distinct component distribution pattern demonstrated that ZGC serves as the primary source of constituents with potential COX-2 binding affinity in SGD, whereas BS provides core active substances that dominate the overall COX-2 inhibitory potency of the formula, revealing distinct chemical complementary distribution of bioactive compounds derived from BS and ZGC.

Macroporous resin fractional separation and quantitative activity validation further characterized the COX-2 inhibitory activity of single herb gradient enrichment fractions. The 40% ethanol eluted fraction of BS, enriched in paeoniflorin and diverse monoterpenoid glycosides, exhibited prominent COX-2 inhibitory activity with an IC_50_ value of 23.8 μg/mL, which was significantly superior to the 80% ethanol fraction of ZGC (IC_50_ = 98.3 μg/mL). The significant discrepancy in inhibitory activity between different herbal fractions fully verified that the excellent anti-inflammatory and analgesic efficacy of SGD does not depend on the pharmacological action of a single monomer compound, but originates from the independent inhibitory activity of various chemical components distributed in different macroporous resin fractions. Moreover, the optimally mixed effective fraction (SGF) displayed stronger inhibitory effect on NO release in LPS-induced RAW 264.7 macrophage models compared with crude SGD extract, further confirming that rational component enrichment can significantly improve the anti-inflammatory bioactivity of herbal extracts, which provides a feasible purification and optimization scheme for the standardized development of SGD effective fractions.

Computational chemistry uncovered the binding mechanism of candidate compounds with COX-2. Molecular docking confirmed all bioactive constituents exhibited strong COX-2 affinity (binding energy < −5.0 kJ/mol); Licoricesaponin H2 had the best binding energy (−78.16 kJ/mol), which shows strong *in silico* binding interaction with COX-2 enzyme. Multi-target molecular docking only predicts that these compounds may interfere with PI3K-Akt, JAK-STAT and other inflammatory signaling pathways, these *in silico* predictions only reflect the multi-binding potential of SGD-derived components, without corresponding functional experimental proof.

Furthermore, 100 ns all-atom molecular dynamics simulations were performed to dynamically verify the stability of target-ligand binding systems. The results confirmed that core active ingredients, including glycyrrhizic acid, licoricesaponin H2, and licorice glycoside B, could form stable binding complexes with potential inflammation-related protein targets (COX-2, EGFR, JAK, PI3K, and STAT3, all binding data are merely computational predictions without cellular validation). These complexes maintained stable structural equilibrium and minor conformational fluctuations throughout the simulation process, accompanied by continuous and stable hydrogen bond interactions with an average hydrogen bond number of 4–7. The dynamic simulation results provide intuitive and reliable molecular evidence for the stable binding and sustained pharmacological activity of SGD active components at the atomic level.

Collectively, this integrated platform combining affinity screening, chemical identification, fraction-activity correlation modeling and multi-dimensional computation provides an efficient universal paradigm to screen bioactive ingredients from complex herbal formulas. This study systematically screens and verifies single constituents with *in vitro* COX-2 inhibitory activity from SGD, and provides analytical and computational clues for its potential *in vitro* anti-inflammatory activity. However, systematic *in vivo* RA model validation is still needed to fully confirm the COX-2-dependent anti-RA mechanism of the complete formula, which offers theoretical reference for the further development of SGD-derived effective fractions as novel natural anti-inflammatory and analgesic lead candidates.

Several limitations exist in the present study. First, only *in vitro* COX-2 enzyme tests of four monomers were completed, without CIA rat *in vivo* validation of the integral formula’s COX-2-related anti-RA efficacy. Second, the PLS spectrum-effect model was built based on separate herb fractions rather than the whole decoction fingerprint. Third, only NO levels were detected in macrophage models, lacking PGE_2_ and COX-2 expression data to directly reflect COX-2 pathway activity. Fourth, COX-1 inhibition assays were absent, so the COX-2 selectivity of tested substances remains unclear. Finally, due to computational cost, the MD simulations were performed with only a single 100 ns trajectory per complex as a preliminary stability assessment, and no MM/PBSA or MM/GBSA binding free energy decomposition calculations were conducted in this study. Therefore, all multi-target computational predictions still require further cellular experimental validation. These computational limitations will be systematically addressed through parallel MD replicates and free energy calculations in our follow-up research, alongside the required *in vivo* pharmacological studies.

## Data Availability

The original contributions presented in the study are included in the article/Supplementary Material. The raw UPLC-MS chromatographic data files are available from the corresponding author upon reasonable request.
