# Supplementary material for: Integrated AUF-UPLC-MS and fraction-activity correlation to identify COX-2-targeted constituents of Shaoyao Gancao decoction
Source: Front Chem. 2026 Sep 21;14:1912431. doi: 10.3389/fchem.2026.1912431 (PMC13635329; doi:10.3389/fchem.2026.1912431)
Supplement: Supplementary file 1 [file DataSheet1.docx]

Supplementary Material

Integrated AUF-UPLC-MS and Fraction-Activity Correlation to Identify COX-2-Targeted Constituents of Shaoyao Gancao Decoction

Liping Li^1,2,†,^ Jun Chi^1,2,†,^ Hejia Wan^1,2,^ Yadi Hou^1,2,^ Bingjie Ren^1,2,^ Yixiao Li^1,2,^ Liping Dai^1,2,*^

^a^ Henan University of Chinese Medicine, Henan Collaborative Innovation Center for Research and Development on the Whole Industry Chain of Yu-Yao, Zhengzhou 450046, China;

^b^ Engineering Technology Research Center for Comprehensive Development and Utilization of Authentic Medicinal Materials in Henan Province, Henan University of Chinese Medicine, Zhengzhou 450046, China

List of Contents

Figure S1. The structures of 19 screened compounds………………….……………………....….2

Figure S2 Base peak chromatograms of blank solvent……….……………………....…....2

**Table	S1** Identification of chemical constituents in SGD.……………….……………………...4

**Figure S3**.Dose–response curves of celecoxib positive control for two separate

COX-2 inhibitory assays..……..........…..........…..........…..........…..........…..........…..........…...20

Figure S4. Summary of fit plots and permutation test results for four PLS regression spectrum-effect models (200 permutations).……..…….....……...……...……...……...….....21

Table	S2. Binding affinity of 19 screened compounds to RA related target proteins …..….......22

Figure S5. Molecular dynamics (MD) simulations………………….……………………….……23

# Abbreviations: SGD, Shaoyao Gancao Decoction; COX‑2, cyclooxygenase‑2; PLS, partial least squares; VIP, variable importance in projection; RA, rheumatoid arthritis

# **Figure S1-1** The structures of 19 screened compounds.

Figure S1-2 The structures of 19 screened compounds.

Figure S2 Base peak chromatograms of blank solvent under positive (A) and negative (B) electrospray ionization modes; no interfering peaks matching SGD components were observed.

**Table S1** Identification of chemical constituents in SGD.

Comparison of MS/MS spectra of paeoniflorin. Upper: authentic reference standard; Lower: corresponding peak from Shaoyao Gancao Decoction. Asterisk (*) in Table S1 denotes compounds confirmed using reference standards; unmarked compounds are tentatively characterized.Source attribution was based on literature reports and the original plant materials.

| **NO.** | **Identification** | **Formula** | ***t*_R_[min]** | **MS [*m/z*]** | **Error [ppm]** | **MS^2^ [*m/z*]** | **Structural types** | **Source** | **Ref** |
| --- | --- | --- | --- | --- | --- | --- | --- | --- | --- |
| 1 | Gallic acid | C_7_H_6_O_5_ | 1.59 | 169.0145 [M−H] ^−^ | 1.33 | 125.0246, 114.0515 | phenolic acids | BS | (13) |
| 2 | 1'-O-Galloylsucrose | C_19_H_26_O_15_ | 1.60 | 493.1207 [M−H] ^−^ | 1.90 | 331.0670, 313.0572, 283.0460, 169.0145 | phenolic acid glycosides | BS | - |
| 3 | Isomer of 1-O-galloyl-β-D-glucose | C_13_H_16_O_10_ | 1.64 | 331.0675 [M−H] ^−^ | 1.56 | 173.1144, 125.0034 | phenolic acid glycosides | BS | - |
| 4 | Plantarenaloside | C_16_H_24_O_9_ | 1.67 | 359.1353 [M−H] ^−^ | 1.65 | 179.0716, 169.0147 | iridoid glycosides | BS | - |
| 5 | Isomer of 1-O-galloyl-β-D-glucose | C_13_H_16_O_10_ | 2.03 | 331.0682 [M−H] ^−^ | 2.27 | 161.1067, 125.0147 | phenolic acids | BS | - |
| 6 | Benzoic acid | C_7_H_6_O_2_ | 2.33 | 123.0402 [M+H] ^+^ | 3.62 | 118.0345, 113.2018, 106.9893, 106.9893, 100.0243, 95.0491, 81.0700, 79.0542, 67.0544 | phenolic acids | BS | (14) |
| 7 | 8-O-Galloyl desbenzoylpaeoniflorin | C_23_H_28_O_14_ | 3.99 | 529.1558 [M+H] ^+^ | 0.58 | 197.0809, 179.0721 | monoterpenoid glycosides | BS | - |
| 8 | Galloyl oxypaeoniflorin | C_30_H_32_O_15_ | 4.08 | 631.5724 [M−H] ^−^ | 2.93 | 479.1127, 379.0411 | monoterpenoid glycosides | BS | (15) |
| 9 | Catechin 7-glucoside | C_21_H_24_O_11_ | 4.15 | 451.1257 [M−H] ^−^ | 2.24 | 289.0751 | dihydroflavonoids | ZGC | - |
| 10 | Isomer of oxypaeoniflorin | C_23_H_28_O_12_ | 4.58 | 495.1526 [M−H] ^−^ | 4.06 | 333.0994,121.0296 | monoterpenoid glycosides | BS | (16) |
| 11 | Paeoniflorin sulfite | C_23_H_28_O_13_S | 4.71 | 543.1187 [M−H] ^−^ | 3.72 | 421.0819,375. 0755, 259.0283 | monoterpenoid glycosides | BS | - |
| 12 | Villosolside | C_16_H_26_O_9_ | 4.91 | 361.1510 [M−H] ^−^ | 1.75 | 179.6686 | iridoid glycosides | BS | - |
| 13 | Hydroxypaeoniflorin | C_23_H_28_O_12_ | 5.08 | 495.1521 [M−H] ^−^ | 4.96 | 465.1408, 165.0561, 137.0248 | monoterpenoid glycosides | BS | (17) |
| 14 | Catechin | C_15_H_14_O_6_ | 5.14 | 289.0723 [M−H] ^−^ | 1.64 | 245.0831, 203.0726 | phenolic acids | BS |  |
| 15 | Hydroxysafflor yellow A | [C](https://pubchem.ncbi.nlm.nih.gov/#query=C27H32O16)_[27](https://pubchem.ncbi.nlm.nih.gov/#query=C27H32O16)_[H](https://pubchem.ncbi.nlm.nih.gov/#query=C27H32O16)_[32](https://pubchem.ncbi.nlm.nih.gov/#query=C27H32O16)_[O](https://pubchem.ncbi.nlm.nih.gov/#query=C27H32O16)_[16](https://pubchem.ncbi.nlm.nih.gov/#query=C27H32O16)_ | 5.21 | 611.1520 [M−H] ^−^ | 0.43 | 281.0679, 165.0563, 137.0250 | flavonoids | ZGC | (18) |
| 16 | Oxypaeoniflorin* | C_23_H_28_O_12_ | 5.58 | 495.1528 [M−H] ^−^ | 2.52 | 345.1200, 333.0976, 281.0678 | monoterpenoid glycosides | BS | (19) |
| 17 | Isomer of neoliquiritin | C_21_H_22_O_9_ | 6.31 | 417.1191 [M−H] ^−^ | −0.13 | 255.0664, 148.0168, 135.0089, 119.0504, 91.0191 | dihydroflavonoid glycosides | ZGC | (20) |
| 18 | Glucoliquiritin apioside | C_32_H_40_O_18_ | 6.52 | 711.2158 [M−H] ^−^ | 3.71 | 549.1619, 255.0663 | dihydroflavonoid glycosides | BS | (20) |
| 19 | Mulberroside F | C_26_H_30_O_14_ | 6.86 | 565.1555[M−H] ^−^ | 2.23 | 271.0619,163.2779,135.0090 | others | - |  |
| 20 | Vicenin-2 | C_27_H_30_O_15_ | 6.89 | 593.1521 [M−H] ^−^ | 2.06 | 473.1118, 353.0650, 297.0763 | flavonoids | BS | (21) |
| 21 | Isomer of mudanpioside E | C_24_H_30_O_13_ | 7.14 | 525.1624 [M−H] ^−^ | 4.21 | 479.1571,283.0828, 121.0297 | monoterpenoid glycosides | BS | (22) |
| 22 | 3-O-β-D-Glucopyranosyl platycodigenin | C_36_H_58_O_12_ | 7.20 | 681.2171 [M−H] ^−^ | 3.62 | 611.1971, 593.1898, 121.0297 | triterpenoid glycosides | BS | - |
| 23 | Isomer of paeoniflorin | C_23_H_28_O_11_ | 7.27 | 481.1630 [M+H] ^+^ | 0.76 | 319.1054, 197.0808, 133.0648, 121.0647, 105.0335 | monoterpenoid glycosides | BS | (15) |
| 24 | Apiin | C_26_H_28_O_14_ | 7.38 | 563.1421 [M−H] ^−^ | 2.06 | 545.1308, 401.0986,325.0720, 211.0481 | flavonoid glycosides | ZGC | (22) |
| 25 | β-D-Glucopyranoside | C_29_H_38_O_16_ | 7.49 | 641.5538 [M−H] ^−^ | 4.43 | 489.1624 | others | BS | - |
| 26 | Isomer of schaftoside | C_26_H_28_O_14_ | 7.66 | 563.1418 [M−H] ^−^ | 2.31 | 503.3003, 473.5012, 297.0767 | flavonoid glycosides | ZGC | - |
| 27 | Paeoniflorin sulfonate | C_23_H_28_O_14_S | 7.93 | 559.1465 [M−H] ^−^ | 0.26 | 399.0617,179.0706,151.0757,133.0651 | monoterpenoid glycosides | BS | - |
| 28 | Paeoniflorin* | C_23_H_28_O_11_ | 8.01 | 479.1557 [M−H] ^−^ | −0.94 | 449.1468, 327.1096, 165.0562, 121.0267 | monoterpenoid glycosides | BS | (19) |
| 29 | Vitamin A | C_20_H_30_O | 8.02 | 285.5100[M−H] ^−^ | 4.64 | 226.5113, 188.9437, 115.3052, 77.0147 | others | BS | - |
| 30 | Isomer of schaftoside | C_26_H_28_O_14_ | 8.11 | 563.1407 [M−H] ^−^ | 0.18 | 503.3003, 443 1010, 383.6348, 353.5500 | flavonoid glycosides | BS | (23) |
| 31 | Asebotin | C_22_H_26_O_10_ | 8.13 | 449.1463 [M−H] ^−^ | 4.62 | 327.1093, 165.0560, 121.0297, 113.0246, 101.0248 | dihydrochalcone glycosides | ZGC | - |
| 32 | Albiflorin | C_23_H_28_O_11_ | 8.59 | 479.1592 [M−H] ^−^ | 2.35 | 276.9591,165.0563,121.0295,87.4760 | monoterpenoid glycosides | BS | (24) |
| 33 | Daidzin | C_21_H_20_O_9_ | 8.61 | 417.1192 [M+H] ^+^ | 3.14 | 336.4864, 123.5150, 110.4180 | isoflavonol glycosides | BS | (25) |
| 34 | Engeletin | C_21_H_22_O_10_ | 8.63 | 433.1140 [M−H] ^−^ | −0.16 | 313.0567, 271.0612, 227.0714, 177.0193, 165.0191, 151.0037, 119.0504, 107.0137, 93.0346 | dihydroflavonol glycosides | ZGC | (26) |
| 35 | Isomer of tetragalloylglucose | C_34_H_28_O_22_ | 8.67 | 787.1015 [M−H] ^−^ | 2.65 | 465.0690, 169.0148, 125.0253 | tannins | BS | - |
| 36 | Isomer of mudanpioside E | C_24_H_30_O_13_ | 8.86 | 525.1626 [M−H] ^−^ | 4.44 | 327.1092,165.0560, 121.0297 | monoterpenoid glycosides | BS | (22) |
| 37 | Isomer of tetragalloylglucose | C_34_H_28_O_22_ | 8.95 | 787.1017 [M−H] ^−^ | 2.90 | 635.0931, 617.0791 | tannins | BS | - |
| 38 | Violanthin | C_27_H_30_O_14_ | 9.01 | 577.1579 [M−H] ^−^ | 4.71 | 559.1465, 487.1255, 457.1158, 383.0784, 353.0676, 341.0691, 325.0724, 297.0776 | flavonoid glycosides | ZGC | (27) |
| 39 | Licraside | C_26_H_30_O_13_ | 9.04 | 549.1621 [M−H] ^−^ | 3.36 | 429.1045,255.0663, 119.0500 | chalcone glycosides | BS | (28) |
| 40 | Neoliquiritin | C_21_H_22_O_9_ | 9.06 | 417.1192 [M−H] ^−^ | 2.05 | 255.0666, 119.0500 | dihydroflavonoid glycosides | ZGC | (27) |
| 41 | Trifolirhizin | C_22_H_22_O_10_ | 9.07 | 447.1295 [M+H] ^+^ | 1.65 | 283.0601, 151.0389,123.0441 | dihydroisoflavone glycosides | BS | - |
| 42 | 5,7^-^Dihydroxyflavanone | C_15_H_12_O_4_ | 9.17 | 257.0809 [M+H] ^+^ | 0.25 | 239.0703, 211.0759, 187.0755, 147.0439, 137.0232, 119.0490, 111.0437, 95.0493 | dihydroflavonoids | ZGC | (29) |
| 43 | Naringin | C_27_H_32_O_14_ | 9.18 | 579.1735 [M−H] ^−^ | 2.88 | 255.0662, 153.0187 | dihydroflavonoids | BS | (30) |
| 44 | Isoliquiritin apioside | C_26_H_30_O_13_ | 9.26 | 549.1620 [M−H] ^−^ | 0.23 | 256.0698, 153.0199, 135.0092 | chalcone glycosides | ZGC | - |
| 45 | Isomer of naringenin^-^7^-^O^-^glucoside | C_21_H_22_O_10_ | 9.31 | 433.1225 [M−H] ^−^ | 2.94 | 313.0565, 227.0713, 177.0193, 151.0037, 145.0295, 119.0502, 93.0345 | dihydroflavonoid glycosides | ZGC | - |
| 46 | Isomer of galloylpaeoniflorin | C_30_H_32_O_15_ | 9.43 | 631.1682 [M−H] ^−^ | 2.52 | 465.1407, 313.0564, 169.0145 | monoterpenoid glycosides | BS | - |
| 47 | Liquiritin apioside | C_26_H_30_O_13_ | 9.43 | 549.1626 [M−H] ^−^ | 2.33 | 256.0663, 135.0086, 119.0500 | dihydroflavonoid glycosides | ZGC | (31) |
| 48 | Isomer of galloylpaeoniflorin | C_30_H_32_O_15_ | 9.74 | 631.1682 [M−H] ^−^ | 2.52 | 465.1407, 313.0564, 169.0145 | monoterpenoid glycosides | BS | - |
| 49 | 6'^-^O^-^Galloyl paeoniflorin | C_30_H_32_O_15_ | 9.79 | 631.1685 [M−H] ^−^ | 4.39 | 399.0942, 375.0729, 313.0571 | monoterpenoid glycosides | BS | (32) |
| 50 | Isomer of galloylpaeoniflorin | C_30_H_32_O_15_ | 10.17 | 631.1673 [M−H] ^−^ | 2.36 | 491.1215, 463.1259,271.0457, 177.0557, 169.0143, 151.0039 | monoterpenoid glycosides | ZGC | (25) |
| 51 | Mudanpioside E | C_24_H_30_O_13_ | 10.29 | 525.1630 [M−H] ^−^ | 2.82 | 495.0038, 479.1559 | monoterpenoid glycosides | BS | - |
| 52 | Trigolin G | C_43_H_46_O_12_ | 10.30 | 594.3514[M+H] ^+^ | 3.71 | 176.0179,162.0148 | diterpenoids | ZGC | - |
| 53 | Pentagalloylglucose | C_41_H_32_O_26_ | 10.31 | 939.1112 [M−H] ^−^ | 1.49 | 465.0723, 169.0147 | tannins | BS | (33) |
| 54 | Diosmetin | C_16_H_12_O_6_ | 10.43 | 299.0561 [M−H] ^−^ | −0.36 | 255.0663, 175.0399, 149.0245, 123.0452 | flavonoids | ZGC | (34) |
| 55 | Isomer of galloylpaeoniflorin | C_30_H_32_O_15_ | 11.01 | 631.1684 [M−H] ^−^ | 4.00 | 463.1305, 313.0581, 169.0147, 121.0299 | monoterpenoid glycosides | BS | - |
| 56 | Isomer of naringenin^-^7^-^O^-^glucoside | C_21_H_22_O_10_ | 11.13 | 435.1293 [M+H] ^+^ | 0.81 | 273.0762,147.0438,81.7160 | dihydroflavonoid glycosides | ZGC | (35) |
| 57 | Hesperetin | C_16_H_14_O_6_ | 11.23 | 301.0723 [M−H] ^−^ | 1.66 | 286.0463 | dihydroflavonoids | BS | (36) |
| 58 | Naringenin chalcone | C_15_H_12_O_5_ | 11.41 | 273.0757 [M+H] ^+^ | −0.47 | 189.0546, 154.0216, 153.0182, 147.0440, 119.0491, 123.0440 | chalcones | ZGC | (36) |
| 59 | Prunin | C_21_H_22_O_10_ | 11.42 | 433.1240 [M−H] ^−^ | 2.66 | 165.0193, 107.0138 | dihydroflavonoid glycosides | BS | - |
| 60 | Tectorigenin | C_16_H_12_O_6_ | 11.43 | 299.0562 [M−H] ^−^ | 0.34 | 284.0328, 240.0425, 212.0474 | isoflavones | BS | (37) |
| 61 | Azelaic acid | C_9_H_16_O_4_ | 11.45 | 189.1129 [M+H] ^+^ | 2.66 | 171.1173,124.9152 | phenolic acids | ZGC | - |
| 62 | Isomer of tectorigenin | C_16_H_12_O_6_ | 11.73 | 299.0565 [M−H] ^−^ | 0.54 | 284.0328, 199.0399, 147.0090 | isoflavones | BS | (38) |
| 63 | Isoferulic acid | C_10_H_10_O_4_ | 11.83 | 195.0654 [M+H] ^+^ | 2.50 | 167.0704,149.0595,95.0492 | phenolic acids | ZGC | (39) |
| 64 | 4^-^Methoxybenzaldehyde | C_8_ H_8_ O_2_ | 11.99 | 137.0597 [M+H] ^+^ | −1.90 | 109.0647, 107.0491, 105.0447, 79.0542,77.0385 | phenolic acids | ZGC | (36) |
| 65 | Glycyroside | C_27_H_30_O_13_ | 12.43 | 561.1622 [M−H] ^−^ | 1.96 | 267.0666, 252.0426 | isoflavonol glycosides | ZGC | (26) |
| 66 | 6''^-^Acetylliquiritin | C_23_H_24_O_10_ | 12.71 | 461.1451 [M+H] ^+^ | 2.32 | 386.1805,299.0917,145.0498 | dihydroflavonoid glycosides | ZGC | (29) |
| 67 | Liquiritin* | C_21_H_22_O_9_ | 12.72 | 417.1199 [M−H] ^−^ | 4.53 | 255.0659, 135.0086, 119.0500 | dihydroflavonoid glycosides | ZGC | (29) |
| 68 | 7,4'^-^Dihydroxyflavone | C_15_H_10_O_4_ | 12.81 | 255.0656 [M+H] ^+^ | 1.43 | 241.8819,182.9001,161.9905 | flavonoids | ZGC | (40) |
| 69 | Ononin | C_22_H_22_O_9_ | 12.82 | 431.1327 [M+H] ^+^ | −2.18 | 332.7977, 269.0806, 260.4074, 190.6722 | isoflavonol glycosides | BS | (41) |
| 70 | Isoliquiritin | C_21_H_22_O_9_ | 12.95 | 417.1195 [M−H] ^−^ | 1.69 | 223.2372, 153.0193, 135.0089, 119.0503, 94.8186, 74.4101 | chalcone glycosides | ZGC | (42) |
| 71 | Neoisoliquiritin | C_21_H_22_O_9_ | 12.99 | 417.1201 [M−H] ^−^ | 2.42 | 255.0662, 135.0086, 119.0500 | chalcone glycosides | ZGC | (37) |
| 72 | Salicylic acid | C_7_H_6_O_3_ | 13.01 | 139.0398 [M+H] ^+^ | −1.33 | 121.10072, 116.9723, 111.9680, 110.0680 | phenolic acids | ZGC | (36) |
| 73 | Licochalcone B | C_16_H_14_O_5_ | 13.03 | 285.0773 [M−H] ^−^ | 1.49 | 150.0326, 133.0265, 121.0293 | chalcone glycosides | ZGC | (43) |
| 74 | Wogonin | C_16_H_12_O_5_ | 13.23 | 285.0773 [M+H] ^+^ | 4.74 | 270.0541, 252.0392, 179.0487 | flavonoids | ZGC | (44) |
| 75 | Licorice glycoside B | C_35_H_36_O_15_ | 13.27 | 695.1996 [M−H] ^−^ | 2.07 | 549.1622, 531.1497, 255.0671, 193.0507 | chalcone glycosides | ZGC | (45) |
| 76 | Licorice glycoside A | C_36_H_38_O_16_ | 13.36 | 727.2242 [M+H] ^+^ | 1.34 | 619.0089,219.0882,177.0554 | chalcone glycosides | ZGC | (46) |
| 77 | Liquiritigenin | C_15_H_12_O_4_ | 13.46 | 255.0666 [M−H] ^−^ | 1.44 | 135.0086, 119.0500, 91.0189 | dihydroflavonoids | ZGC | (47) |
| 78 | Isomer of liquiritin | C_21_H_22_O_9_ | 13.47 | 419.1344 [M+H] ^+^ | −0.50 | 257.0809,176.2296,147.0447,90.3582 | dihydroflavonoid glycosides | ZGC | (48) |
| 79 | Chrysoeriol | C_16_H_12_O_6_ | 13.85 | 299.0568 [M−H] ^−^ | 4.04 | 599.1218, 300.0587, 133.5926, 110.5404, 78.9594, 63.3142 | flavonoids | ZGC | (36) |
| 80 | 7,7-Dihydroebuloside | C_21_H_34_O_10_ | 14.54 | 447.2237 [M+H] ^+^ | 2.23 | 285.0759,227.0534,172.8004 | iridoid glycosides | ZGC | - |
| 81 | Atractyloside A | C_21_H_36_O_10_ | 14.94 | 447.2246 [M−H] ^−^ | 2.14 | 285.1624 | sesquiterpenoid glycoside | ZGC | (24) |
| 82 | Palmitic acid | C_16_H_32_O_2_ | 15.06 | 257.2477 [M+H] ^+^ | 0.79 | 239.0715,137.0235 | fatty acids | ZGC | (48) |
| 83 | Jasmonic acid | C_12_H_18_O_3_ | 15.56 | 211.1327 [M+H] ^+^ | −1.22 | 165.1274, 151.1106, 123.0804 | fatty acids | BS/ ZGC | - |
| 84 | Uralsaponin F | C_44_H_64_O_19_ | 15.74 | 895.3983 [M−H] ^−^ | 2.51 | 545.34766, 527.33716 | triterpenoid saponins | ZGC | (29) |
| 85 | Licoricesaponin G2 | C_42_H_62_O_17_ | 15.88 | 839.4059 [M+H] ^+^ | 2.70 | 487.3417, 469.3306 | triterpenoid saponins | ZGC | (29) |
| 86 | Benzoylpaeoniflorin* | C_30_H_32_O_12_ | 15.96 | 583.1838 [M−H] ^−^ | 2.84 | 553.1725, 165.0558 | monoterpenoid glycosides | ZGC | (36) |
| 87 | Pinobanksin | C_15_H_12_O_5_ | 15.99 | 273.0761 [M+H] ^+^ | 1.42 | 137.0600,123.0452 | flavonol glycosides | ZGC | (49) |
| 88 | Benzoylalbiflorin | C_30_H_32_O_12_ | 16.00 | 583.1839 [M−H] ^−^ | −0.27 | 535.1588, 431.1356 | monoterpenoid glycosides | ZGC | - |
| 89 | Mudanpioside J | C_31_H_34_O_14_ | 16.03 | 631.2012 [M+H] ^+^ | -1.33 | 437.3432,347.3389,261.6612,122.6147 | monoterpenoid glycosides | ZGC | (50) |
| 90 | Apigenin | C_15_H_10_O_5_ | 16.03 | 269.0816 [M−H] ^−^ | −2.32 | 254.0573, 240.1416, 237.0564, 209.0612, 175.0405, 161.0248, 149.10612, 120.0222, 116.9312 | flavonoids | ZGC | (51) |
| 91 | Isomer of retrochalcone | C_16_H_14_O_4_ | 16.07 | 271.0969[M+H] ^+^ | 1.60 | 229.0861,194.3889,177.0554,121.0287,107.0494 | chalcones | ZGC | (46) |
| 92 | Glycitein | C_16_H_12_O_5_ | 16.18 | 283.0612 [M−H] ^−^ | 2.76 | 268.0377, 240.0421, 239.0341 | isoflavones | ZGC | - |
| 93 | Isomer of benzoylpaeoniflorin | C_30_H_32_O_12_ | 16.36 | 585.1811 [M+H] ^+^ | −0.56 | 319.1176, 249.0757, 197.0808, 151.0753, 133.0647, 105.0334 | monoterpenoid glycosides | ZGC | (34) |
| 94 | Licoricesaponin A3 | C_48_H_72_O_21_ | 16.37 | 985.4655 [M+H] ^+^ | 2.03 | 615.3908,453.3373,435.3269,357.2440 | triterpenoid saponins | ZGC | (52) |
| 95 | Isomer of benzoylpaeoniflorin | C_30_H_32_O_12_ | 16.39 | 585.1978 [M+H] ^+^ | −1.03 | 375.7625,267.0876,105.0336 | monoterpenoid glycosides | BS | (19) |
| 96 | Calycosin | C_16_H_12_O_5_ | 16.41 | 283.0619 [M−H] ^−^ | 0.43 | 268.0387, 239.0691, 224.0496, 214.8823, 197.9122, 166.7873 | isoflavones | ZGC | (36) |
| 97 | Isomer of retrochalcone | C_16_H_14_O_4_ | 16.52 | 271.0961 [M+H] ^+^ | −1.53 | 229.0857, 177.0554, 121.0282, 105.8188, 63.8835 | chalcones | ZGC | - |
| 98 | 22-Acetoxyglycyrrhizin | C_44_H_64_O_18_ | 16.86 | 879.4029 [M−H] ^−^ | 2.06 | 351.05396 | triterpenoid saponins | ZGC | - |
| 99 | Glabrolide | C_30_H_44_O_4_ | 17.12 | 469.3323 [M+H] ^+^ | 1.10 | 451.3215, 405.3156 | triterpenoids | ZGC | (53) |
| 100 | Benzoyloxypaeoniflorin | C_30_H_32_O_13_ | 17.13 | 601.1975 [M+H] ^+^ | 2.91 | 121.0301 | monoterpenoid glycosides | BS | (16) |
| 101 | 5,7-Dihydroxyflavanone | C_15_H_12_O_4_ | 17.59 | 257.0806 [M+H] ^+^ | −1.03 | 239.0700, 229.0854, 211.0751, 197.0617, 183.0805, 155.0337, 147.0439, 137.0232, 119.0488 | dihydroflavonoids | ZGC | (54) |
| 102 | Isoglabrolide | C_30_H_44_O_4_ | 18.10 | 469.3314 [M+H] ^+^ | 0.24 | 451.3205, 405.3154 | triterpenoids | ZGC | (55) |
| 103 | Glabranin | C_20_H_20_O_4_ | 18. 14 | 323.8787 [M−H] ^−^ | 1.45 | 322.8615, 305.8768, 298.7294, 254.9278, 238.9045, 186.9399, 121.3382, 78.9594, 61.9886 | isoflavones | ZGC | (36) |
| 104 | Formononetin | C_16_H_12_O_4_ | 18.21 | 269.0806 [M+H] ^+^ | −0.99 | 254.0567,237.0543, 226.0621, 213.0909, 172.9345, 136.0159, 118.0412, 107.0490, 81.0698 | isoflavones | ZGC | - |
| 105 | Isoformononetin | C_16_H_12_O_4_ | 18.65 | 267.0667 [M−H] ^−^ | 1.58 | 252.0426, 195.0447 | isoflavones | ZGC | - |
| 106 | Hydroxyglycyrrhizic acid | C_42_H_62_O_17_ | 18.87 | 839.4051 [M−H] ^−^ | 0.32 | 645.3628, 487.3416, 469.3310, 451.3203 | triterpenoids | ZGC | (29) |
| 107 | Chiisanoside | C_48_H_74_O_19_ | 19.34 | 953.4748 [M−H] ^−^ | −0.63 | 205.0716, 113.0244 | triterpenoids | ZGC | (29) |
| 108 | Glycyrrhizic acid* | C_42_H_62_O_16_ | 19.82 | 823.4110 [M+H] ^+^ | −1.61 | 759.3924, 471.3479, 425.3461, 351.0576 | triterpenoid saponins | ZGC | - |
| 109 | 8-Desbenzoylpaeoniflorin | C_16_H_24_O_10_ | 19.44 | 375.1304 [M−H] ^−^ | 1.83 | 195.1395 | monoterpenoid glycosides | ZGC | (36) |
| 110 | Uralsaponin B | C_42_H_62_O_16_ | 19.86 | 823.4107 [M+H] ^+^ | −2.01 | 647.3786, 592.4236, 471.3470, 453.3362, 435.3251 | triterpenoid saponins | ZGC | - |
| 111 | Licoricesaponin E2 | C_42_H_60_O_16_ | 19.97 | 819.3814 [M−H] ^−^ | 2.08 | 351.05466, 193.03468, 113.02438 | triterpenoid saponins | ZGC | (55) |
| 112 | Glycyrrhetinic acid | C_30_H_46_O_4_ | 19.97 | 471.3462 [M+H] ^+^ | −1.35 | 453.3383, 407.3310, 317.2119, 235.1697, 189.1636, 175.1493, 149.1318, 135.1168, 121.1019, 95.0861 | triterpenoids | ZGC | (56) |
| 113 | 3-Oxoglycyrrhetinic acid | C_30_H_44_O_4_ | 20.06 | 469.331 [M+H] ^+^ | −0.53 | 451.3215, 433.3082, 405.3149, 315.1970, 297.1839, 261.1486, 233.1531, 217.1579, 175.1481, 135.1169, 107.0856, 93.0700 | triterpenoids | ZGC | (55) |
| 114 | Topazolin | C_21_H_20_O_6_ | 20.06 | 369.1329 [M+H] ^+^ | −0.99 | 351.1224, 332.7684,229.0871, 193.0491, 165.0555, 151.2486, 117.4685, 63.7429 | flavonoids | BS | - |
| 115 | Licoricesaponin H2 | C_42_H_62_O_16_ | 20.12 | 823.4107 [M+H] ^+^ | −3.32 | 692.8439, 647.3777, 471.3465, 453.3361, 435.3264 | triterpenoid saponins | ZGC | (57) |
| 116 | Medicarpin | C_16_H_14_O_4_ | 20.35 | 271.0946 [M+H] ^+^ | −6.81 | 257.2648, 202.8855, 161.0602, 142.3487, 137.0597, 123.0441 | flavonoids | BS | (36) |
| 117 | Licoricesaponin K2 | C_42_H_62_O_16_ | 20.99 | 821.3976 [M−H] ^−^ | 2.23 | 351.0524, 193.0345, 113.0243 | triterpenoid saponins | ZGC | - |
| 118 | Gancaonin N | C_21_H_20_O_6_ | 21.01 | 369.1334 [M+H] ^+^ | −4.7 | 321.3629, 313.0703, 285.0774, 271.0600, 243.0652, 188.2318, 150.6719, 124.6523 | isoflavones | BS | (58) |
| 119 | Apioglycyrrhizin | C_41_H_62_O_14_ | 21.21 | 777.4082 [M−H] ^−^ | 2.68 | 647.3784, 471.3481, 453.3360 | triterpenoid saponins | ZGC | (29) |
| 120 | Glycycoumarin | C_21_H_20_O_6_ | 21.76 | 367.1187 [M+H] ^+^ | 1.49 | 313.0710, 285.0755, 270.0521 | coumarins | BS | (59) |
| 121 | 8^−^Prenyleriodictyol | C_20_H_20_O_6_ | 22.68 | 355.1193 [M−H] ^−^ | 4.80 | 203.0718, 151.0405 | dihydroflavonoid glycosides | ZGC | (29) |
| 122 | Licoricesaponin J2 | C_42_H_64_O_16_ | 22.69 | 823.4139 [M−H] ^−^ | −1.61 | 752.5027, 351.0557, 289.0578, 193.0538 | triterpenoid saponins | ZGC | - |
| 123 | Licoricesaponin C2 | C_42_H_62_O_15_ | 22.71 | 805.403 [M−H] ^−^ | 2.58 | 453.3437, 193.0342 | triterpenoid saponins | ZGC | (29) |
| 124 | Licoisoflavanone | C_20_H_18_O_6_ | 22.95 | 355.1569 [M+H] ^+^ | 4.20 | 299.0922, 287.0914, 215.1060, 191.1066, 153.0546, 149.0596, 95.8412 | isoflavones | BS | (60) |
| 125 | Licoisoflavanone A | C_20_H_18_O_6_ | 22.99 | 355.1170[M+H] ^+^ | −1.73 | 299.0551, 287.0549, 271.0608, 130.3187, 69.0699 | isoflavones | ZGC | (36) |
| 126 | Benzophenone | C_13_ H_10_ O | 23.00 | 183.0804 [M+H] ^+^ | −0.66 | 105.0335, 77.0384 | others | BS | (61) |
| 127 | Glyasperin C | C_21_H_24_O_5_ | 23.15 | 355.1555 [M−H] ^−^ | 1.55 | 135.0450, 121.0293 | isoflavones | BS | (62) |
| 128 | Semilicoisoflavone B | C_20_H_16_O_6_ | 23.59 | 353.0512 [M+H] ^+^ | 0.51 | 257.0549, 139.0042 | isoflavones | BS | (36) |
| 129 | ViceninⅡ | C_27_H_30_O_15_ | 23.61 | 593.1511 [M−H] ^−^ | 1.83 | 575.1379, 503.1185, 473.1189, 455.0984, 425.0884, 395.0759, 383.0774, 365.0661, 353.0666, 325.0715, 297.0764, 242.9439 | flavonoid glycosides | ZGC | (63) |
| 130 | Isolicoflavonol | C_20_H_18_O_6_ | 23.81 | 355.1167 [M+H] ^+^ | −2.58 | 343.3910, 308.9004, 299.0548, 269.0918, 257.4044, 165.0175 | flavonols | ZGC | (60) |
| 131 | Xanthohumol | C_21_H_22_O_5_ | 23.93 | 353.1391 [M+H] ^+^ | 2.29 | 297.0764,267.0659,199.0749,146.7249 | chalcones | BS | (64) |
| 132 | Licochalcone A | C_21_H_22_O_4_ | 24.17 | 339.159 [M+H] ^+^ | 1.22 | 297.1485, 283.0984, 271.0964, 191.1066, 177.0546, 121.0284, 107.0491 | chalcones | BS | (65) |
| 133 | Isomer of bis (4^-^ethylbenzylidene) sorbitol | C_24_H_30_O_6_ | 24.53 | 415.2108 [M+H] ^+^ | −0.52 | 133.0645, 119.0854, 97.0290, 91.0542 | others | BS | (36) |
| 134 | Glabrone | C_20_H_16_O_5_ | 24.58 | 337.1068 [M+H] ^+^ | −0.89 | 319.0968, 295.0601, 239.0699, 201.0550, 137.0232, 81.0698 | isoflavones | BS | (66) |
| 135 | Pinocembrin | C_15_H_12_O_4_ | 25.42 | 257.0805 [M+H] ^+^ | −1.17 | 239.0703, 211.0746, 147.0440, 137.0232, 127.0390 | dihydroflavonoids | BS | (67) |
| 136 | Gancaonin E | C_25_H_28_O_6_ | 26.99 | 425.1951 [M+H] ^+^ | −1.80 | 387.7839, 369.1317, 351.1210, 313.0702, 301.0702, 295.0597, 277.1666, 221.1166, 179.0334, 175.0388, 165.0542, 147.0437,139.0388, 135.0438, 117.9547 | dihydroflavonoids | BS | (36) |
| 137 | Glepidotin B | C_20_H_20_O_5_ | 27.00 | 339.1241 [M−H] ^−^ | 3.98 | 219.0663, 133.0662, 119.0501, 72.1626 | dihydroflavonols | BS | (68) |
| 138 | Uralenol | C_20_H_18_O_7_ | 28.38 | 371.1846 [M+H] ^+^ | −0.69 | 315.1226, 303.1224, 261.1490, 235.1327, 193.0858, 181.0859, 167.0702, 149.0596, 137.0599, 123.0439, 110.1229, 69.0698 | flavonols | BS | (36) |
| 139 | Isomer of morusin | C_25_H_24_O_6_ | 28.63 | 421.1629 [M+H] ^+^ | −0.99 | 379.1158, 365.1016,347.0910,337.1094,311.0550, 201.0944, 183.0282, 165.0179, 165.0179, 109.0652 | flavonoids | ZGC | (69) |
| 140 | 3,5^-^Di^-^tert^-^butyl^-^4^-^hydroxybenzaldehyde | C_15_ H_22_ O_2_ | 28.99 | 235.1626 [M+H] ^+^ | −0.48 | 219.1380, 180.1100, 179.1066, 163.0753, 123.0440, 57.0698 | others | BS | - |
| 141 | Sigmoidin B | C_20_H_20_O_6_ | 29.08 | 355.1193 [M−H] ^−^ | 4.80 | 203.0718, 151.0405 | dihydroflavonoids | BS | - |
| 142 | Glicoricone | C_21_H_20_O_6_ | 29.28 | 369.1331 [M+H] ^+^ | −6.19 | 351.1226, 313.0697, 191.1066, 179.0345, 135.0440, 123.0440, 75.3352 | isoflavones | BS | (70) |
| 143 | Tetramethylcurcumin | C_25_H_28_O_6_ | 29.34 | 425.1948 [M+H] ^+^ | −2.46 | 369.1321, 351.1250, 313.0703, 301.0705, 233.0804, 191.1063, 175.0388, 139.0388, 79.7418, 69.0698 | others | BS | (36) |
| 144 | (±)13^-^Coriolic acid | C_19_H_32_O_3_ | 29.46 | 299.2278 [M−H] ^−^ | –0.29 | 277.2175, 195.1390, 183.1386, 171.1017, 113.0974, 59.0143 | fatty acids | ZGC | - |
| 145 | Isomer of morusin | C_25_H_24_O_6_ | 29.37 | 421.1629 [M+H] ^+^ | −3.95 | 365.1022, 323.0549, 180.6127, 165.0180, 151.6461, 111.4947 | flavonoids | BS | - |
| 146 | Glyasperin A | C_25_H_26_O_6_ | 29.57 | 423.1792 [M+H] ^+^ | −2.47 | 367.1194, 359.5831, 340.9129, 311.0537, 256.0832, 216.1821, 201.0914, 189.0906 | flavonols | BS | - |
| 147 | Isomer of glyasperin | C_25_H_26_O_6_ | 29.64 | 423.1783 [M+H] ^+^ | −4.50 | 367.1167, 355.1160, 311.0546 | flavonoids | BS | (36) |
| 148 | Isomer of glyasperin | C_25_H_26_O_6_ | 30.69 | 423.1798 [M+H] ^+^ | −1.03 | 367.1171, 349.1064, 311.0547, 299.0549, 283.0578, 165.0187 | flavonoids | ZGC | (36) |
| 149 | Dibutyl phthalate | C_16_H_22_O_4_ | 30.77 | 279.1596 [M+H] ^+^ | 1.87 | 149.0237,102.7475,57.0669 | others | BS | (36) |
| 150 | Isomer of morusin | C_25_H_24_O_6_ | 30.86 | 421.1642 [M+H] ^+^ | −0.99 | 365.1016, 347.0903, 201.0920, 183.0292, 165.0183, 141.0183 | flavonoids | BS | - |
| 151 | Ursolic acid | C_30_ H_48_ O_3_ | 34.51 | 455.3564 [M−H] ^−^ | 2.92 | - | triterpenoids | ZGC | (71) |
| 152 | Hexadecanamide | C_16_ H_33_ N O | 36.12 | ^−^256.2635 [M+H] ^+^ | −0.25 | 130.1227, 116.1070, 102.0913, 100.0756, 88.0756, 74.0600, 69.0698 | fatty acids | BS | (72) |
| 153 | Medrogestone | C_23_H_32_O_2_ | 37.86 | 339.2334 [M−H] ^−^ | 4.72 | 199.8515,163.1131,113.3552 | triterpenoids | ZGC | (73) |

(References cited in the “Ref.” column are numbered consistently with the main text reference list. Please refer to the References section of the main manuscript for full citation details.)

A

B

Figure S3.Dose–response curves of celecoxib positive control for two separate COX-2 inhibitory assays. (A) Celecoxib COX-2 inhibition curve determined via prostaglandin E₂ EIA assay (IC₅₀ = 28.7 nM); (B) Celecoxib COX-2 inhibition curve determined via fluorescent COX-2 assay (IC₅₀ = 30.4 nM).

D2

D1

C2

C1

B1

A2

A1

Figure S4. Summary of fit plots and permutation test results for four PLS regression spectrum-effect models (200 permutations). (A1,A2) ZGC, negative ion mode; (B1,B2) ZGC, positive ion mode; (C1,C2) BS, negative ion mode; (D1,D2) BS, positive ion mode. Summary of fit plots were applied to select optimal components based on 7-fold cross-validation. Permutation tests were used to assess model overfitting risk.

B2

**Table S****2** Binding affinity of 19 screened compounds to RA related target proteins.

| **Structural types** | **Name** | **binding energy（-kJ/mol）** | | | | | | | | |
| --- | --- | --- | --- | --- | --- | --- | --- | --- | --- | --- |
|  |  | **COX-2** | **NF-κB** | **PI3K** | **AKT** | **STAT3** | **JAK** | **HSP90AA1** | **SRC** | **EGFR** |
| monoterpenoid glycosides | Paeoniflorin | 22.48 | - | 50.19 | 35.24 | 58.80 | 53.46 | - | 48.97 | 44.25 |
| triterpenoid saponins | Glycyrrhizic acid | 64.08 | - | 85.50 | 65.44 | - | - | - | - | 60.34 |
| chalcone glycosides | Licorice glycoside B | 56.92 | - | 79.60 | 58.45 | 84.11 | 81.38 | - | 67.80 | 76.25 |
| triterpenoid saponins | Licoricesaponin H2 | 78.16 | - | 86.29 | 60.65 | - | - | - | - | 54.97 |
| triterpenoid saponins | Licoricesaponin G2 | 71.84 | - | 82.25 | 59.23 | - | - | - | - | 54.95 |
| triterpenoids | Glycyrrhetinic acid | 33.16 | - | 55.98 | 45.32 | 24.81 | 25.35 | - | 39.46 | 35.12 |
| triterpenoid saponins | Uralsaponin B | 68.65 | - | 83.87 | 61.87 | - | - | - | - | 64.03 |
| triterpenoids | Glabrolide | 24.92 | - | 47.46 | 33.29 | - | - | - | - | 30.16 |
| triterpenoids | 3-Oxoglycyrrhetinic acid | 37.04 | - | 53.39 | 36.64 | 23.96 | 29.20 | - | - | 31.44 |
| flavonoid glycosides | Violanthin | 46.79 | - | 74.31 | 55.03 | 85.34 | 65.48 | - | 74.29 | 77.18 |
| monoterpenoid glycosides | Albiflorin | 28.82 | - | 52.62 | 39.04 | 54.21 | 62.19 | - | 48.12 | 49.87 |
| dihydroflavonoid glycosides | Liquiritin apioside | 38.03 | - | 66.51 | 47.34 | 68.87 | 70.17 | - | 54.81 | 60.08 |
| dihydroflavonoid glycosides | Liquiritin | 28.68 | 37.85 | 52.23 | 41.74 | 55.87 | 57.21 | - | 48.04 | 46.95 |
| dihydroflavonoids | Liquiritigenin | 13.29 | 37.49 | 31.51 | 23.45 | 37.30 | 36.63 | 22.48 | 32.98 | 31.81 |
| dihydroflavonol glycosides | Engeletin | 25.76 | 33.87 | 53.45 | 37.80 | 58.83 | 58.26 | 64.08 | 50.33 | 46.94 |
| triterpenoids | Isoglabrolide | 29.10 | - | 50.08 | 36.60 | 30.06 | 26.18 |  | 32.36 | 31.54 |
| isoflavonol glycosides | Ononin | 31.16 | 28.78 | 50.78 | 41.40 | 55.25 | 58.12 |  | 46.00 | 42.91 |
| chalcone glycosides | Isoliquiritin | 30.72 | 45.20 | 59.35 | 43.88 | 59.25 | 60.76 |  | 47.76 | 55.68 |
| fatty acids | Jasmonic acid | 26.35 | 41.10 | 38.45 | 27.33 | 31.59 | 35.54 | 34.26 | 27.14 | 34.75 |
| diarylpyrazole | Celecoxib | 18.76 |  |  |  |  |  |  |  |  |

C

B

A

Figure S5. Multiple trajectory parameters derived from 100 ns all-atom molecular dynamics simulations of eight target-ligand complexes. A-C: Time evolution of solvent accessible surface area (SASA), intermolecular hydrogen bond number and radius of gyration (Rg).D-E: Backbone root-mean-square deviation (RMSD) and root-mean-square fluctuation (RMSF) of amino acid residues. Each subplot corresponds to an individual protein-ligand complex. RMSD evaluates global structural stability; RMSF characterizes residue flexibility; SASA and (Rg) reflect protein compactness in aqueous solution; hydrogen bond trajectories illustrate dynamic intermolecular interactions between target proteins and ligands.

E

D
